# A Novel Epigenetic Silencing Pathway Involving the Highly Conserved 5’-3’ Exoribonuclease Dhp1/Rat1/Xrn2 in *Schizosaccharomyces pombe*

**DOI:** 10.1371/journal.pgen.1005873

**Published:** 2016-02-18

**Authors:** James Franklin Tucker, Corina Ohle, Géza Schermann, Katja Bendrin, Wei Zhang, Tamás Fischer, Ke Zhang

**Affiliations:** 1 Department of Biology, Wake Forest University, Winston-Salem, North Carolina, United States of America; 2 Biochemistry Center (BZH), Heidelberg University, Heidelberg, Germany; 3 Department of Microbiology-Immunology, Feinberg School of Medicine, Northwestern University, Chicago, Illinois, United States of America; University of California San Francisco, UNITED STATES

## Abstract

Epigenetic gene silencing plays a critical role in regulating gene expression and contributes to organismal development and cell fate acquisition in eukaryotes. In fission yeast, *Schizosaccharomyces pombe*, heterochromatin-associated gene silencing is known to be mediated by RNA processing pathways including RNA interference (RNAi) and a 3’-5’ exoribonuclease complex, the exosome. Here, we report a new RNA-processing pathway that contributes to epigenetic gene silencing and assembly of heterochromatin mediated by 5’-3’ exoribonuclease Dhp1/Rat1/Xrn2. Dhp1 mutation causes defective gene silencing both at peri-centromeric regions and at the silent mating type locus. Intriguingly, mutation in either of the two well-characterized Dhp1-interacting proteins, the Din1 pyrophosphohydrolase or the Rhn1 transcription termination factor, does not result in silencing defects at the main heterochromatic regions. We demonstrate that Dhp1 interacts with heterochromatic factors and is essential in the sequential steps of establishing silencing in a manner independent of both RNAi and the exosome. Genomic and genetic analyses suggest that Dhp1 is involved in post-transcriptional silencing of repetitive regions through its RNA processing activity. The results describe the unexpected role of Dhp1/Rat1/Xrn2 in chromatin-based silencing and elucidate how various RNA-processing pathways, acting together or independently, contribute to epigenetic regulation of the eukaryotic genome.

## Author Summary

Epigenetic mechanisms regulate when, where, and how an organism uses the genetic information stored in its genome. They are essential to many cellular processes, such as the regulation of gene expression, genome organization, and cell-fate determination. They also govern growth, development, and ultimately human health. Heterochromatin constitutes silenced chromatic domains, in which gene silencing occurs through epigenetic mechanisms. RNA processing pathways, such as RNA interference (RNAi) and the exosome, are known to mediate the silencing of genes via degradation of unwanted or aberrant transcripts. In this study, we describe a new RNA processing mechanism in epigenetic silencing using fission yeast, a premier model for studying these processes. With genetic, cell biology, and genomic approaches, we uncovered a previously unrecognized function of Dhp1, a highly conserved 5’-3’ exoribonuclease and ortholog of budding yeast Rat1 and metazoan Xrn2. We show that Dhp1 mediates a novel RNA processing mechanism in epigenetic silencing which occurs independently of both RNAi and the exosome. Our results clarify how multiple RNA processing pathways are involved in the regulation of eukaryotic gene expression and chromatin organization.

## Introduction

In eukaryotic cells, DNA coils around histones to form nucleosomes, which are packaged into chromatin. Various post-translational modifications (PTMs) of histones, histone variants, and nucleosome remodeling factors confer distinct chromatin states on genes and facilitate the organization of large chromatin tracts into domains [1–3]. Euchromatic domains (euchromatin) contain actively transcribed genes and are enriched with hyperacetylated histones, while heterochromatic domains (heterochromatin) contain highly repetitive elements that are transcriptionally silenced and are associated with hypoacetylated histones [4–7]. In addition to its primary role in transcriptional gene silencing, heterochromatin is crucial for centromere-mediated chromosome segregation, cell fate determination, and the silencing of repetitive DNA elements [4]. In fission yeast, *Schizosaccharomyces pombe* (*S*. *pombe*), its formation requires both histone hypoacetylation and histone H3 methylation at lysine 9 (H3K9me), which provides a binding site for HP1 family proteins [8–10]. Heterochromatin is first nucleated at specific repetitive loci and subsequently spread for up to hundreds of kilobases (kb) into surrounding regions [4, 11, 12]. Once established, these silenced heterochromatic domains are heritable, and can be stably maintained through successive cell divisions [4, 13, 14].

Epigenetic silencing includes both transcriptional (TGS) and post-transcriptional gene silencing (PTGS). In general, heterochromatin limits the access of RNA polymerase II (RNAPII) machinery to the DNA template and can therefore mediate transcriptional gene silencing (TGS) by preventing unwanted transcription from a given genomic region [15, 16]. PTGS employs RNA processing machinery to rapidly degrade nascent RNAs to repress gene expression or to protect the genome from foreign genetic elements such as retroviral RNA or transposable DNA [17–20]. RNA processing machineries ensure the maturation and packaging of RNAs from longer precursors into mRNA/protein particles (mRNPs) before they are exported to the cytoplasm for translation [21]. Most of these processing events, such as addition of the 5’ cap, removal of introns, and polyadenylation at the 3’ end, occur while the RNA is still attached to RNAPII and chromatin, and are therefore referred to as transcription-coupled RNA processing events [22, 23]. For example, RNA endocleavage at a polyadenylation (polyA) site is commonly required for transcriptional termination because the 5’ to 3’ exoribonucleolysis of the exposed 3’ fragment downstream of the polyA site facilitates RNAPII release from chromatin [24, 25]. Besides their roles in RNA maturation and RNAPII termination, RNA-processing enzymes act as quality control systems, screening partly or fully transcribed products and degrading abnormal RNAs [22, 26]. RNA processing pathways play an active role in epigenetic silencing, especially PTGS [27], as many nuclear processes rely on the fine balance between RNA maturation and destruction to regulate gene expression [28].

The best understood RNA processing pathway in PTGS is the RNA interference (RNAi)-mediated formation of heterochromatin at centromeric regions in *S*. *pombe* [27, 29, 30]. In RNAi, RNAPII transcripts originating from repetitive DNA regions are converted to double-stranded RNA by the RNA-Dependent RNA Polymerase Complex (RDRC) [31]. They are then processed by Dicer into small interfering RNAs (siRNAs) [32, 33] and loaded onto the Argonaute-containing RNA Induced Transcriptional Silencing (RITS) complex, which targets the repeat regions through the homology of the siRNA sequence [34]. RITS associates with chromatin by direct interaction with H3K9me [35], then recruits the Clr4 complex to initiate chromatin remodeling [36–39]. Recently, several studies reported an RNAi-independent RNA-processing pathway in heterochromatin assembly at the centromeric region and some heterochromatic islands in euchromatic regions [40–43]. This new pathway is mediated by the exosome complex, which degrades unwanted RNAs via its 3’-5’ exoribonuclease activity [26]. Although both RNAi and exosome pathways are RNA-mediated and involved in processing long noncoding RNAs (ncRNAs) into small RNAs, how the exosome pathway contributes to heterochromatin assembly is not well understood. In addition, it is not known whether other RNAi-independent RNA processing pathways participate in epigenetic silencing.

Here we report a new epigenetic silencing pathway involving Dhp1, a conserved 5’→3’ exoribonuclease, the ortholog of budding yeast Rat1 and metazoan Xrn2 known to promote termination of RNA polymerase II (RNAPII) transcription [44–46]. We show that Dhp1-mediated heterochromatic silencing is independent of Din1, an ortholog of budding yeast Rai1 that has been shown to stabilize Dhp1/Rat1 exoribonuclease activity [47]. In addition to maintenance of gene silencing, Dhp1 contributes to *de novo* establishment of heterochromatin at the centromeres and the silent mating type region. It also plays a role in the transcriptional-dependent spreading of heterochromatin. Importantly, Dhp1 interacts with heterochromatic factors and its catalytic activity is required for its role in silencing. Further genetic analyses indicate that Dhp1 operates in a distinct pathway parallel to RNAi and the exosome to mediate heterochromatic gene silencing. Finally, RNAPII localization and transcriptome-wide maps of RNAs associated with RNAPII revealed that Dhp1 likely acts at the post-transcriptional level to affect gene silencing. We propose that, in addition to RNAi and exosomes, Dhp1 constitutes a distinct RNA-processing pathway that enforces post-transcriptional gene silencing across the fission yeast transcriptome.

## Results

### *dhp1-1* but not *din1Δ* cells exhibit silencing defects at centromeric regions and the mating type locus

**Fig 1:**
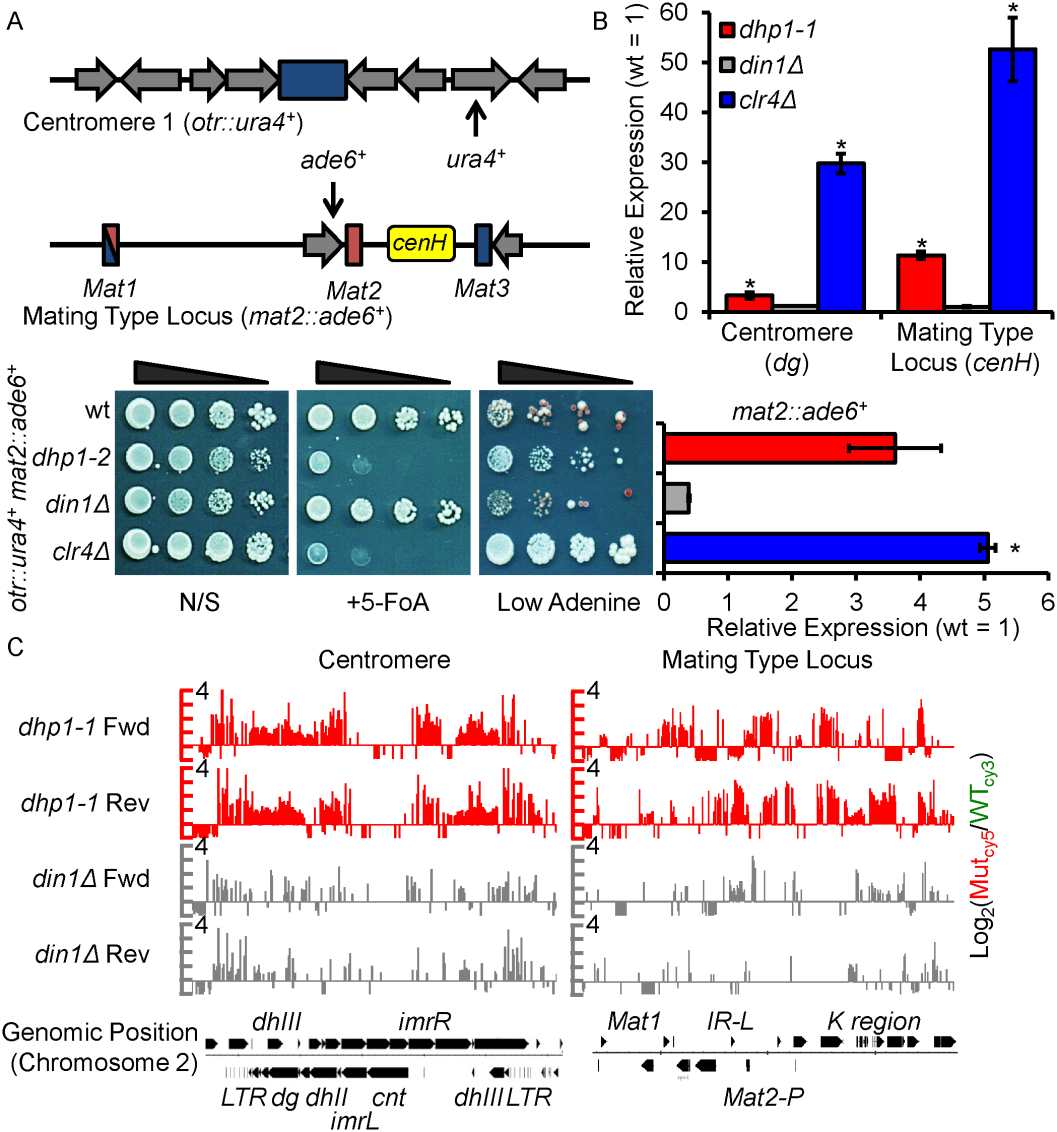
*dhp1* but not *din1* mutants exhibit epigenetic silencing defects. (A) Above: diagram showing the sites of insertion for reporter genes *ura4*^*+*^ and *ade6*^*+*^ in an outer terminal repeat region (*otr*) of centromere 1 and the mating type locus (*mat*), respectively. Below: serial dilution demonstrating the expression of *otr*∷*ura4*^*+*^ (on counter-selective 5-FoA-containing media) and *mat2*∷*ade6*^*+*^ (on low adenine media) reporter genes in wildtype (wt), *dhp1-2*, and *din1Δ*. N/S, non-selective rich media. (B) Above: qRT-PCR reveals relative levels of transcripts produced from within the *dg* and *cenH* regions and shows increased expression in *dhp1-1* compared with wt or *din1Δ*. Below: qRT-PCR shows relative expression of the *mat2*∷*ade6*^*+*^ reporter. * *p* ≤ 0.05 as determined by Student’s *t* test comparing the indicated sample values with wt values. Error bars represent s.e.m. (C) Expression profiling shows substantial upregulation of repeat transcripts across the mating type locus and the centromere in *dhp1-1* cells, but not *din1Δ* cells. The map of the genomic region is displayed below the figure (Fwd, forward strand; Rev, reverse strand). Chromosome positions were annotated based on the released 2007 *pombe* genome.

Dhp1/Xrn2 is an essential gene required for transcriptional termination and RNA quality control [22, 45, 46]. A recent study reported that impairment of transcription termination is sufficient to induce the formation of heterochromatin at protein-coding genes by *trans*-acting siRNAs in *S*. *pombe* [48]. However, it is not clear whether impaired transcription termination would alter epigenetic silencing at the major heterochromatic regions such as centromeric regions and the mating type locus. We therefore tested whether silencing at these regions is affected in *dhp1* mutant cells. Because its loss is lethal, we utilized a conditional temperature-sensitive (*ts*) allele, *dhp1-1*, which codes a truncated carboxyl-terminal form of Dhp1 that is partially replaced by a *ura4*^*+*^ transgene and is lethal at 37°C (*dhp1-1*>>*ura4*^+^) [47]. We generated an independent *dhp1-2* mutant, which carries the same carboxylic terminal truncation but is replaced with a nourseothricin resistance gene (NatN2) (S1 Table). This allele has a less severe *ts* phenotype compared to *dhp1-1*, and is not lethal at 37°C (S1A Fig). *dhp1-2* is not fused with *ura4*^*+*^, allowing us to investigate the silencing of the centromeric region by analyzing the expression of a *ura4*^*+*^ reporter gene inserted at the outer centromeric region (*otr*∷ *ura4*^*+*^) (Fig 1A, top panel). Wildtype cells carrying the reporter *otr*∷*ura4*^*+*^ grew well on counter-selective medium containing 5-Fluoroorotic Acid (5-FoA) indicating *otr*∷*ura4*^*+*^ was silenced (Fig 1A, bottom panel). The growth of *dhp1-2* was greatly inhibited in the presence of 5-FoA indicating defective silencing of the reporter gene. Surprisingly, *din1*-null (*din1Δ*) cells do not have a severe growth or centromeric silencing defect, suggesting that Dhp1 is involved in a silencing pathway separate from its Din1-related activity (Fig 1A, bottom panel). To further examine the observed silencing defect, an *ade6*^*+*^ reporter gene was inserted into the silenced mating type region (Fig 1A, top panel). We can easily observe the silencing status of the *ade6*^*+*^ based on color; cells that cannot express the normal level of the reporter gene accumulate a red pigment due to blocked adenine biosynthesis. In wildtype cells, the reporter gene is silenced by heterochromatin, resulting in red/sectored colonies on low adenine media at 30°C. Cells lacking Clr4, the sole histone H3K9 methyltransferase in *S*. *pombe* [49], form white colonies due to loss of heterochromatin (Fig 1A). Similar to *clr4Δ*, *dhp1-2* but not *din1*-null (*din1Δ*) cells form white colonies, indicating a silencing defect at the mating type locus unique to the *dhp1* mutant. Since *dhp1-1* has a more severe silencing defect than *dhp1-2* as evaluated by silencing assay and quantitative Reverse Transcription Polymerase Chain Reaction (qRT-PCR) (S1 Fig), the rest of our studies focused on using the *dhp1-1* allele. We next assessed whether loss of reporter gene silencing in the *dhp1* mutant is correlated with the expression of heterochromatic repeats. Transcript analysis by qRT-PCR revealed substantially unregulated expression of repeats associated with pericentromeric heterochromatin and the silent mating type locus in *dhp1* but not *din1* mutant cells (Fig 1B). Further analysis by expression profiling using a tiling microarray on both DNA strands showed increased expression throughout both heterochromatic regions in *dhp1-1*, well above the increase observed in *din1Δ* cells (Fig 1C). These results indicate that Dhp1 plays a previously unrecognized, Din1-independent function in epigenetic silencing.

To avoid potential pleotropic effects caused by *dhp1* mutation, we performed all our experiments at 30°C. This is a permissive temperature for *dhp1-1*, in which silencing defects but no obvious growth deficiency are observed (Fig 1A), suggesting that most of transcription-related functions of Dhp1 are retained. In addition, we carefully analyzed the transcriptional levels of all known heterochromatic factors in *dhp1-1* cells using expression data. All coding transcripts of these proteins are affected less than 1.6-fold (a typical threshold difference for microarray data) compared to that of wildtype (S2 Table). Further, a plasmid-borne wildtype copy of *dhp1*^*+*^ rescues the *ts* phenotype of *dhp1-1* (S2A Fig) and a diploid heterozygous strain carrying a wildtype and a *dhp1-1* allele showed the same phenotype as a wildtype diploid strain (S2B and S2C Fig), demonstrating that *dhp1-1* has no dominant negative effects. Altogether, these findings suggest that the loss of heterochromatic silencing in the *dhp1* mutant is likely a direct consequence of impaired function of Dhp1 at heterochromatin rather than reduced transcription of heterochromatic factors.

### Both *dhp1-1* and *din1Δ* cells are defective in RNAPII transcription termination

The silencing defect in *dhp1-1* is unexpected because impaired transcription termination would reduce RNAPII transcription and the subsequent release of the RNA from the site of transcription, which may enhance the assembly of heterochromatin through induction of RNA-mediated chromatin modification such as H3K9 methylation (H3K9me) [48, 50]. In addition, many reported Dhp1 functions are associated with Din1, which contributes to the generation of the proper substrates for Dhp1’s exoribonuclease activity [46, 51]. Because we did not observe a silencing defect in *din1Δ* cells, we wondered whether Din1, like Dhp1 is involved in transcription termination. According to the “Torpedo model” [24, 25], Dhp1-mediated exonucleolysis of the cleaved 3’ fragment downstream of the mRNA polyA site facilitates RNAPII release from chromatin. Deficiency in Dhp1/Din1 will cause RNAs to accumulate at the 3’ end of genes, due to an RNAPII transcription termination defect [24, 25]. To confirm this reported role of Dhp1/Din1, we analyzed the transcriptomes of *dhp1-1* and *din1Δ* cells at euchromatic regions. We detected a genome-wide increase of RNA levels at the 3’ end of genes compared to wildtype in both mutants, with a larger fraction of genes exhibiting transcription termination defects in *dhp1-1* (S3 Fig). While the role of Dhp1 is more dominant than that of Din1, these results support earlier studies indicating that both Dhp1 and Din1 participate in RNAPII transcription termination.

As an interacting protein of Rat1/Xrn2, Rtt103 also contributes to transcription termination in yeast and humans [52–54]. Rhn1, the *S*. *pombe* ortholog of Rtt103, has a reported role in the suppression of meiotic mRNAs during vegetative growth [54]. However, whether it plays a role in heterochromatic silencing has not been reported. To further examine whether defective transcription termination is crucial for Dhp1-mediated epigenetic silencing, we compared the expression of repeat elements in wildtype and *rhn1Δ* cells using qRT-PCR and found that, like Din1, loss of Rhn1 did not cause a silencing defect (S4 Fig). Taken together, our data argue that Dhp1 plays a novel role in epigenetic silencing, which cannot be explained by its established function in transcription termination.

### Mutation of *dhp1* results in impaired chromosome segregation and heterochromatin defects

**Fig 2:**
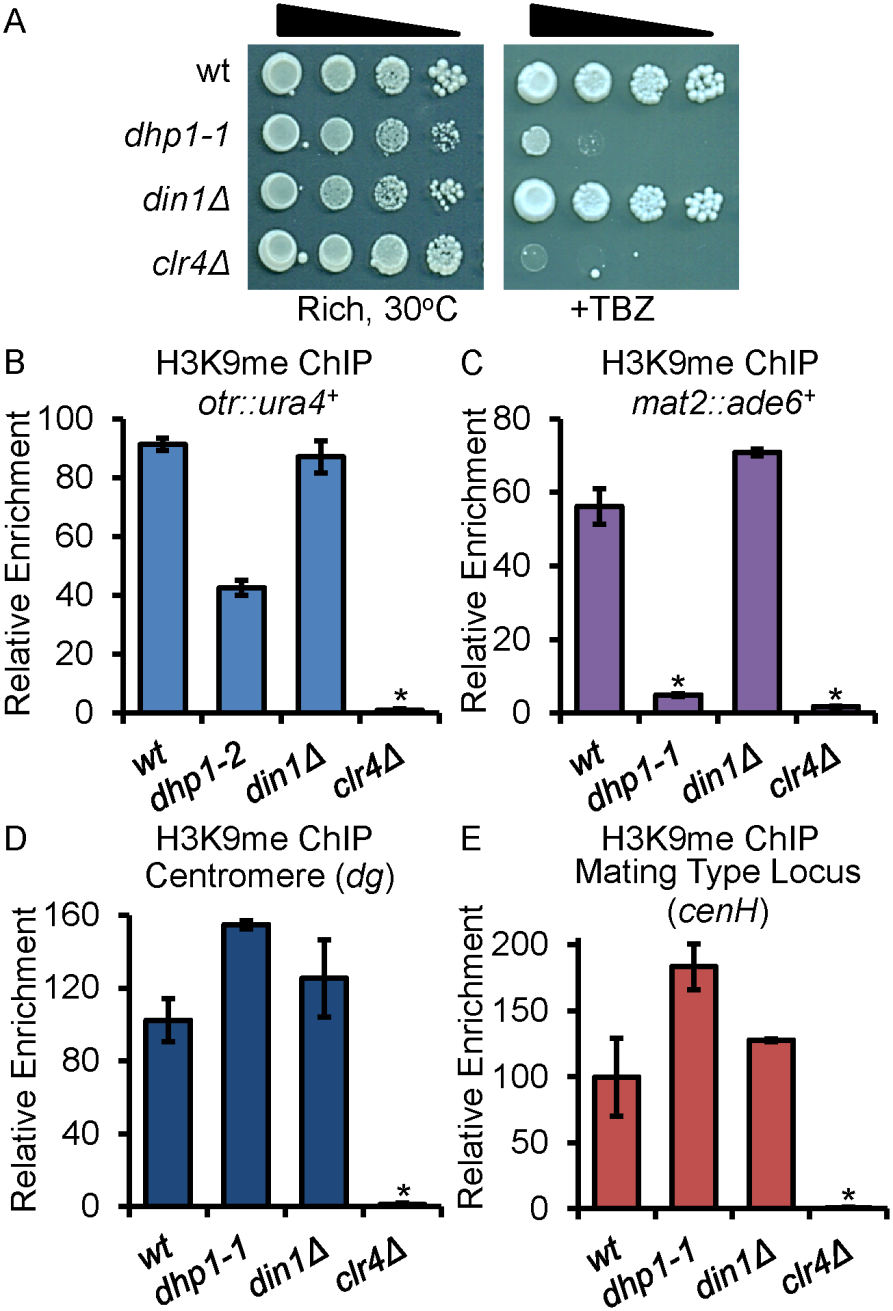
Defective heterochromatin in *dhp1* mutants is marked by reduced H3K9 methylation at reporter genes. (A) Ten-fold serial dilution on media containing 20 μg/ml TBZ demonstrates chromosome segregation defects in *dhp1-1* mutants. (B-C) Chromatin immunoprecipitation (ChIP) performed with an anti-H3K9me_2_ antibody shows reduced H3K9me_2_ enrichment in *dhp1-1* at the reporter genes located at pericentromeric region (*otr*, B) and *mat2* (C). (D-E) The same reduction was not observed at the centromeric (D) and mating type locus (E) repeat regions by H3K9me2 ChIP. Relative enrichment (normalized to input) gives the approximate ratio between H3K9me_2_ levels on the indicated heterochromatic regions versus the euchromatic control *leu1*^*+*^. * *p* ≤ 0.05 as determined by Student’s *t* test comparing the indicated sample values with wt values.

Cells with mutations in factors that contribute to epigenetic silencing often exhibit defects in chromosome segregation as determined by their sensitivity to the microtubule-destabilizing drug thiabendazole (TBZ) [55]. Because heterochromatin formation has been linked to centromere function in various organisms including *S*. *pombe* [56–58], we tested whether the *dhp1* mutants are sensitive to TBZ, which would indicate impaired function of centromeric heterochromatin, resulting in a chromosome segregation defect. As expected, deletion of *clr4* abolishes heterochromatin and causes severe TBZ sensitivity (Fig 2A). Our assay clearly shows that *dhp1*, but not *din1*, mutant cells are sensitive to TBZ, suggesting a chromosome segregation defect specific to *dhp1* mutants (Fig 2A). To further examine the role of Dhp1 in chromosome segregation, we sporulated wildtype and mutant *h*^*90*^ strains to follow the segregation of chromosomes in tetrads using a fluorescence-based analysis (S5 Fig). To sporulate, two haploid cells with opposite mating types conjugate to form a zygote which then enters meiosis. During meiosis, cells undergo two consecutive rounds of chromosome segregation. A normal meiosis results in an ascus in which each of four spores contain relatively equal amounts of DNA (DAPI dots). Abnormal meiotic segregation within a tetrad will show an uneven distribution of DAPI staining in each spore, resulting in less than or greater than four dots. We found that meiotic chromosome segregation is severely perturbed in the *dhp1-1*, but not in *din1Δ* cells, with nearly 50% of tetrads containing abnormal numbers of DAPI dots (≤ 3 or ≥ 5) (S5 Fig).

To determine whether the chromosome segregation defect seen in the *dhp1* mutant is linked to its role in epigenetic silencing at major heterochromatic domains, we assessed the status of H3K9me-associated heterochromatin by a Chromatin-Immunoprecipitation (ChIP) assay. Although no reduction of H3K9me_2_ was seen at the endogenous repetitive regions (Fig 2D and 2E), the levels of H3K9me_2_ at the reporter genes embedded in these regions were substantially reduced at these loci in cells deficient in *dhp1* (Fig 2B and 2C). Loss of *din1* has no negative effect on the enrichment of the H3K9me mark at either the endogenous repetitive regions or the reporter genes (Fig 2B–2E). These results suggest that Dhp1’s role in chromosome segregation is linked to its requirement to maintain functional heterochromatin at the centromeres.

### Dhp1 but not Din1 interacts with heterochromatic proteins

We next wondered whether Dhp1 interacts with heterochromatic proteins, which would support a direct role of Dhp1 in facilitating heterochromatin assembly. We purified Dhp1 and Din1 through two-step affinity purification (S6 Fig) and identified the co-purified proteins by mass spectrometry analysis (S3 Table). Strains used for purification carry a functional Dhp1 or Din1 fused with FTP, a modified TAP tag comprising a protein A motif and a FLAG tag separated by a TEV protease cleavage site. Since Dhp1 and Din1 are associated with transcribing RNAs and chromatin, we performed all purifications in the presence of Benzonase to avoid indirect protein-protein interactions mediated by nucleic acids. Din1 is the major Dhp1-interacting protein as recovered Din1 peptides were found to be approximately 50% as abundant as those of the bait protein, Dhp1. As expected, Dhp1 also co-purified with many RNAPII-related factors, consistent with its role in transcriptional termination. In particular, it is associated with several heterochromatic proteins, including Clr4 methyltransferase complex (ClrC) subunit Rik1 and exosome subunit Rrp6. These data are consistent with interactions recently identified in a parallel study [59]. Notably, these heterochromatic proteins were not present in those fractions when Din1 was used as the bait, supporting a distinct role of Dhp1 in heterochromatic formation. Our data indicates that Dhp1 interacts with heterochromatic proteins and is likely directly involved in heterochromatin assembly.

### Dhp1 is required for *de novo* establishment of silencing at heterochromatic repeat regions

**Fig 3:**
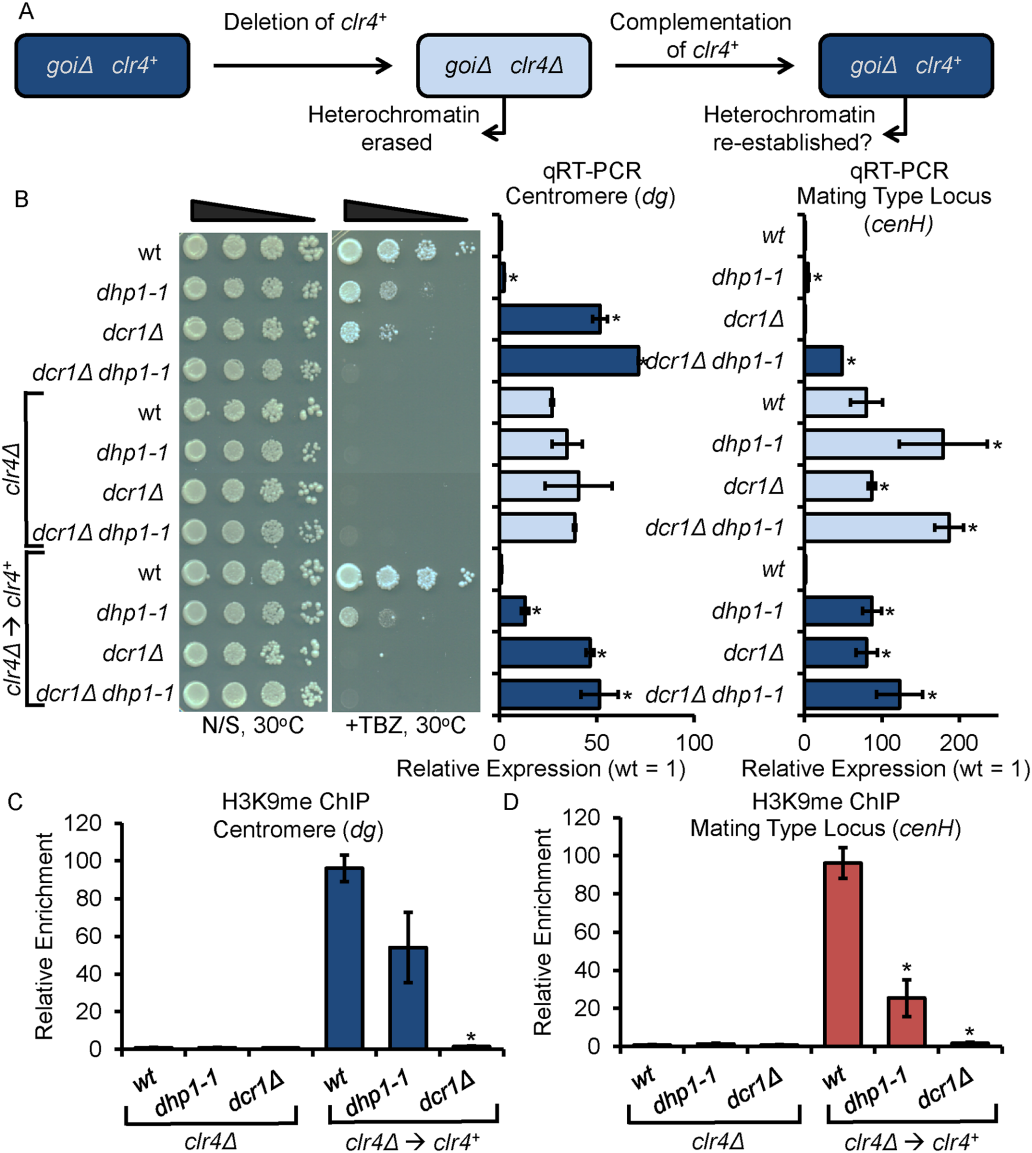
Dhp1 is required for efficient *de novo* establishment of heterochromatin. (A) A role for a gene of interest (*goi*) in *de novo* establishment of heterochromatin is identified by deleting or mutating the *goi* in the *clr4Δ* background in which heterochromatin domains will be erased (derepressed). Re-introduction of functional *clr4*^*+*^ allows the formation of repressive chromatin provided that the *goi* is dispensable for heterochromatin establishment. (B) Left: dilution assay on TBZ shows completely resumed repression in the wt control following the re-introduction of *clr4*^*+*^, but only partially resumed repression in *dhp1* mutants. Right: qRT-PCR showing relative *dg* and *cenH* transcript levels demonstrates the loss of repression in all strains with the *clr4Δ* background and completely resumed repression in wt cells only following complementation of *clr4*^*+*^. Slight resumption of repression of *dg* and *cenH* repeats is observed in *dhp1-1* mutants. (C-D) H3K9me_2_ qChIPs were performed using indicated strains. Relative enrichments at indicated genomic regions were normalized to *leu1*^*+*^. * *p* ≤ 0.05 as determined by Student’s *t* test comparing the indicated sample values with the corresponding wt values (for example: *clr4Δ→ clr4*^*+*^ mutants were compared to *clr4Δ→ clr4*^*+*^ wt) for qRT-PCR and qChIP.

Multiple pathways are utilized to initiate epigenetic silencing including both RNA and DNA sequence-dependent mechanisms [9, 60, 61]. Several studies have shown that in *S*. *pombe*, both RNAi and the exosome contribute to the initiation of silencing at the centromere by processing RNAs transcribed from repetitive regions [13, 41, 61]. We next sought to determine whether Dhp1 also contributes to this process through examination of reporter gene expression following the reintroduction of functional *clr4*^*+*^ into *dhp1-1 clr4Δ* double mutant cells (Fig 3A). Deletion of *clr4* results in the abolition of H3K9me and the loss of heterochromatin. However, reintroduction of functional *clr4*^*+*^ is sufficient for *de novo* heterochromatin formation as previously reported [50] (Fig 3B). One of the key members in RNAi machinery, Dcr1, is the sole Dicer-family endoribonuclease in *S*. *pombe* [62]. *dcr1Δ* cells lose the ability to initiate heterochromatin formation *de novo* at repeat regions [13]. Consistent with previous findings, silencing at the centromeric region cannot be efficiently established without Dcr1 [13]. Reintroduction of *clr4*^*+*^ into *clr4Δ* cells shows a complete alleviation of TBZ sensitivity, while *clr4*^*+*^ reintroduction into *dcr1Δ clr4Δ* double mutant cells has no effect (Fig 3B). Additionally, complementation of *clr4*^*+*^ has little effect on the relative expression of centromeric- and mating type locus-specific repeats in *dcr1Δ clr4Δ* cells, however silencing of these repeats is fully resumed in *clr4Δ* single mutants (Fig 3B). Reintroduction of *clr4*^*+*^ in *dhp1-1 clr4Δ* double mutant cells partially resumed the silencing at the centromeric region as indicated by qRT-PCR (Fig 3B). At the mating type locus, silencing is barely restored in *dhp1-1 clr4Δ*, having 10-fold more expression than *dhp1-1* alone (Fig 3B). H3K9me_2_ ChIP analysis further demonstrated that without functional Dhp1, H3K9me_2_ is partially re-established at the centromeric region, but only a low level of H3K9me_2_ can be found at the mating type locus after complementation of *clr4*^*+*^ (Fig 3C and 3D). These results indicate that Dhp1 is essential for efficient *de novo* heterochromatin assembly at peri-centromeres and the silent mating type locus.

### Dhp1 facilitates the transcription-related spreading of H3K9me

Spreading of heterochromatin from the nucleation sites enables the establishment of a heterochromatin domain spanning many kbps [12]. Although the mechanism is poorly understood, it depends on the oligomerization of chromatin modifiers such as HP1 and Tas3, a RITS component, and the actions of Swi6-recruited histone deacetylases (HDACs) on adjacent nucleosomes [63–66]. While the polymerization of chromatin modifiers indeed constitutes a major part of heterochromatin spreading, a role for RNAPII in transcription-mediated spreading is currently being explored. The effect of spreading on the silencing of reporter genes inserted into centromeric repeat regions has been shown to vary with position relative to the RNAPII promoter; reporter genes downstream of the promoter are more effectively silenced than those inserted upstream [67, 68]. While the molecular details remain unclear, transcription-mediated spreading appears to require transcription of the 3’ untranslated region as well as degradation of these transcripts by RNAi machinery [67].

**Fig 4:**
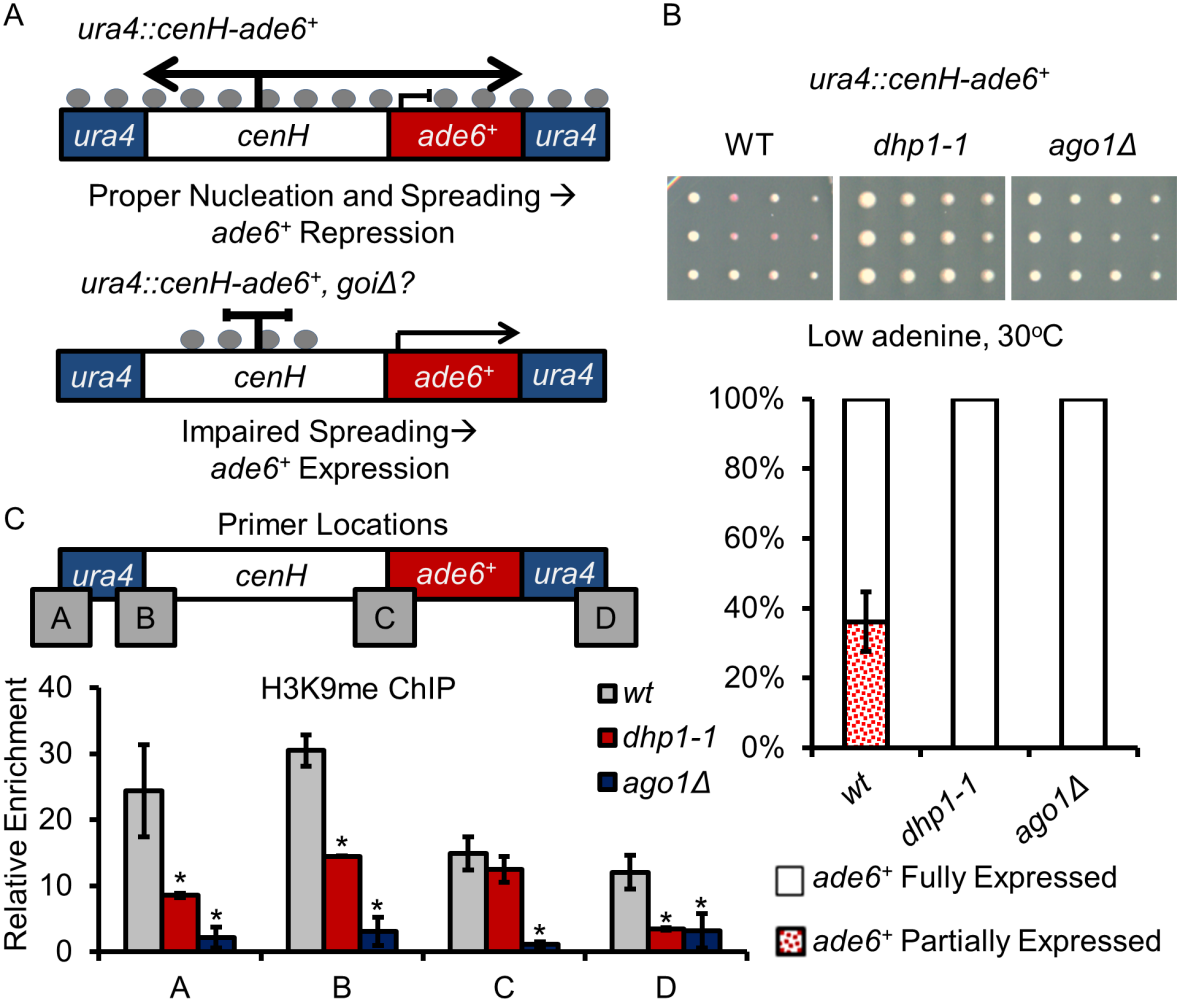
Dhp1 contributes to heterochromatin spreading. (A) Insertion of the *cenH* nucleation site adjacent to the *ade6*^*+*^ reporter gene in an ectopic euchromatic locus (*ura4*^*+*^) will result in the silencing of the reporter gene through heterochromatin spreading. Gray circles: H3K9me. (B) Above, representative images of grid-based assays used to calculate the percentage of cells exhibiting distinct *ade6*^*+*^ expression on low adenine medium. Below: 100% stacked column graph represents the observed phenotypes of these colonies. (C) H3K9me_2_ qChIP shows relative enrichment at the *ade6*^*+*^ reporter gene and its surrounding regions normalized to *leu1*^*+*^. The genomic positions for oligos used for qPCR are indicated by gray boxes. * *p* ≤ 0.05 as determined by Student’s *t* test comparing the indicated sample values with wt values.

Decreased enrichment of H3K9me at the reporter genes in *dhp1-1* suggests that Dhp1 may be involved in the spreading of H3K9me (Fig 2B and 2C). Because of its role in transcriptional termination, we next examined whether Dhp1 is required for RNAi-dependent spreading of heterochromatin, which partially relies on RNAPII transcription [68]. To this end, we adopted a spreading assay [13, 69] (Fig 4A). Nucleation of heterochromatin at *cenH* is dependent on RNAi and the *cenH* sequence itself [13, 69]. Inserting the *cenH* sequence into a euchromatic locus causes ectopic establishment followed by spreading of heterochromatin and subsequent silencing of proximal genes [13]. By coupling ectopic *cenH* with an adjacent reporter gene (*ade6*^*+*^), we can directly observe the effects of spreading of silencing to the proximal reporter gene (Fig 4A). In the wildtype background, about 35% of cells form pink/sectoring colonies, indicating the spreading of heterochromatin assembled at ectopic *cenH* to the *ade6*^*+*^ reporter gene. Cells lacking *ago1*, a critical factor in RNAi form white colonies at 100% efficiency showing that the *ade6*^*+*^ reporter gene cannot be silenced. This is consistent with previous results indicating that RNAi machinery is required for the transcriptional-dependent spreading of heterochromatin assembled at *cenH* region [13, 68]. Similar to *ago1Δ*, no pink colonies were formed in *dhp1-1* background (Fig 4B). H3K9me_2_ ChIP using multiple primers along *ade6*^*+*^ and its surrounding regions shows moderate reduction of this heterochromatic mark in *dhp1-1* cells compared to wildtype cells (Fig 4C). Altogether, these results suggest that Dhp1 plays a role in the transcription-related spreading of the H3K9me mark.

### Dhp1 contributes to the maintenance of heterochromatic silencing at the mating type locus

**Fig 5:**
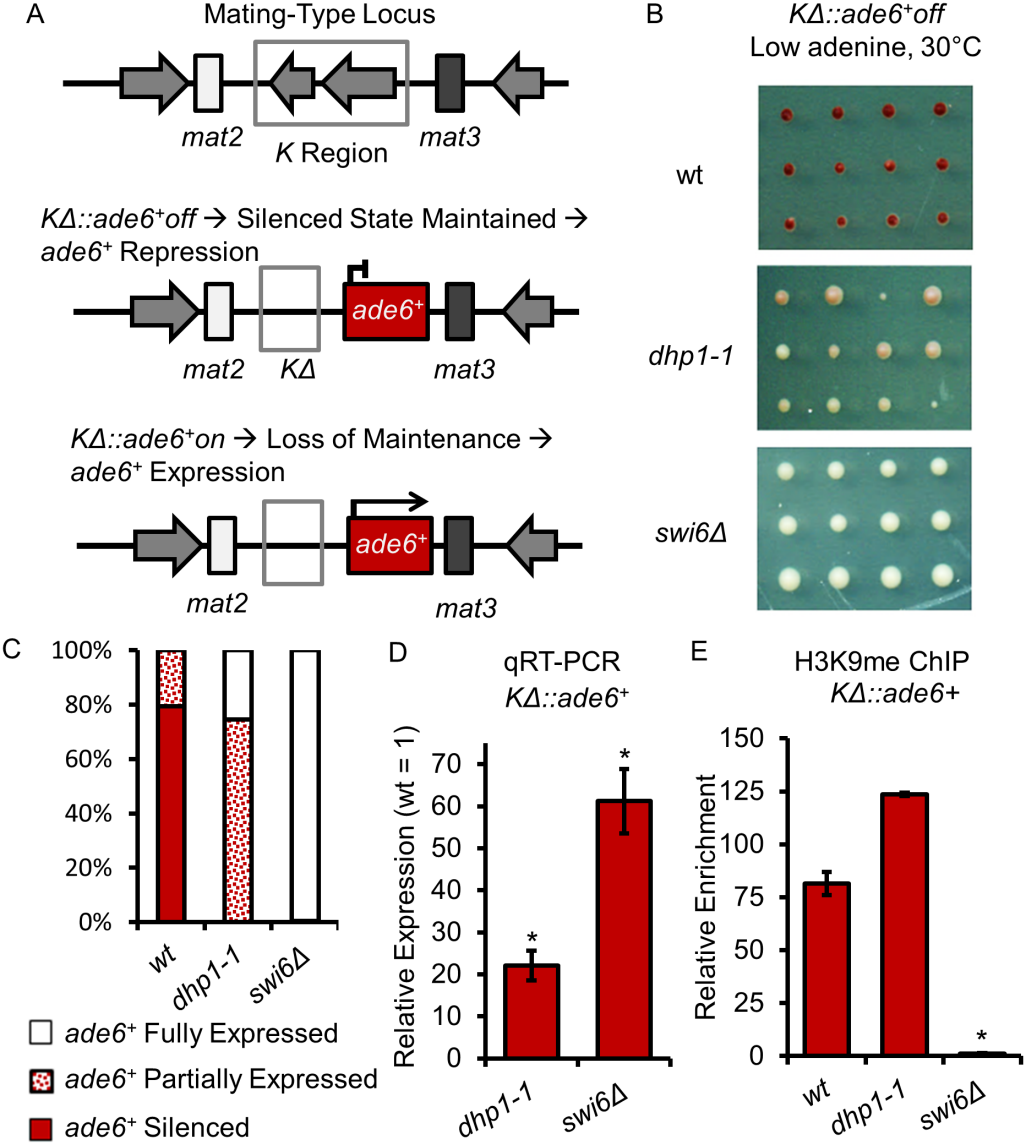
Dhp1 is required for effective heterochromatin maintenance. (A) A map of the mating type locus shows the *cenH*-containing *K* region, a nucleation site from which repressive chromatin is initiated and spread. After the initial establishment of heterochromatin, deletion of the *K* region does not affect the chromatin structure and the reporter gene (*ade6*^*+*^) remains silenced due to maintenance. Disruption of maintenance results in derepression of the reporter gene. (B) Representative images of grid-based assays from which the percent of colonies grouped by *ade6*^*+*^ expression were calculated. (C) Graph displays the percent of colonies grouped by relative expression of *ade6*^*+*^ as visually determined by colony color. (D) qRT-PCR shows the relative expression compared to wt of the *ade6*^*+*^ reporter gene in *dhp1-1* and *swi6Δ*. (E) H3K9me_2_ qChIP was carried out on the indicated strains. * *p* ≤ 0.05 as determined by Student’s *t* test comparing the indicated sample values with the wt values for qRT-PCR and qChIP.

While RNAi is critical to the establishment and spreading of heterochromatin, it is dispensable for the maintenance of a previously assembled chromatin state at the mating type locus and sub-telomeric regions [64, 70]. To investigate the role of Dhp1 in heterochromatin maintenance, we introduced the *dhp1* mutation into cells that lack part of the *K* region in the mating type locus, but continue to repress a proximal *ade6*^*+*^ reporter gene (*KΔ*∷*ade6*^*+*^off) (Fig 5A). Deleting the *K* region results in loss of heterochromatin establishment within the mating type locus, but because heterochromatin is stably inherited through cell division, derepression is rarely seen without concomitant loss of the maintenance machinery [71]. Loss of maintenance machinery will result in derepression of *ade6*^*+*^ (*KΔ*∷*ade6*^*+*^off will switch to *KΔ*∷*ade6*^*+*^on). While the molecular mechanisms which mediate maintenance remain unclear, Clr4 and Swi6 have been implicated [13, 38, 72]. We detected partial loss of repression of *ade6*^*+*^ in *dhp1-1*, an intermediate phenotype between wildtype and *swi6Δ* (Fig 5B and 5C). In the wildtype background, nearly 80% of cells form dark red colonies and only about 20% of cells form pink colonies. Unlike *swi6Δ*, which form 100% white colonies, about 75% of *dhp1-1* cells form pink colonies, although no dark red colonies were ever observed. We further analyzed the *ade6*^*+*^ RNA level by qRT-PCR (Fig 5D). Indeed, we observed a more than 20-fold increase the amount of *ade6*^*+*^ transcripts in *dhp1-1* cells compared to that of wildtype cells. Consistent with previous studies, loss of Swi6 abolishes the enrichment of H3K9me_2_ at *KΔ*∷*ade6*^*+*^ (Fig 5E)[72]. Interestingly, mutation of *dhp1* does not reduce this histone modification at the same region (Fig 5E), suggesting that Dhp1 plays a role in effective maintenance of epigenetic silencing downstream of H3K9me.

### Dhp1 mediates epigenetic silencing independent of RNAi and the exosome

**Fig 6:**
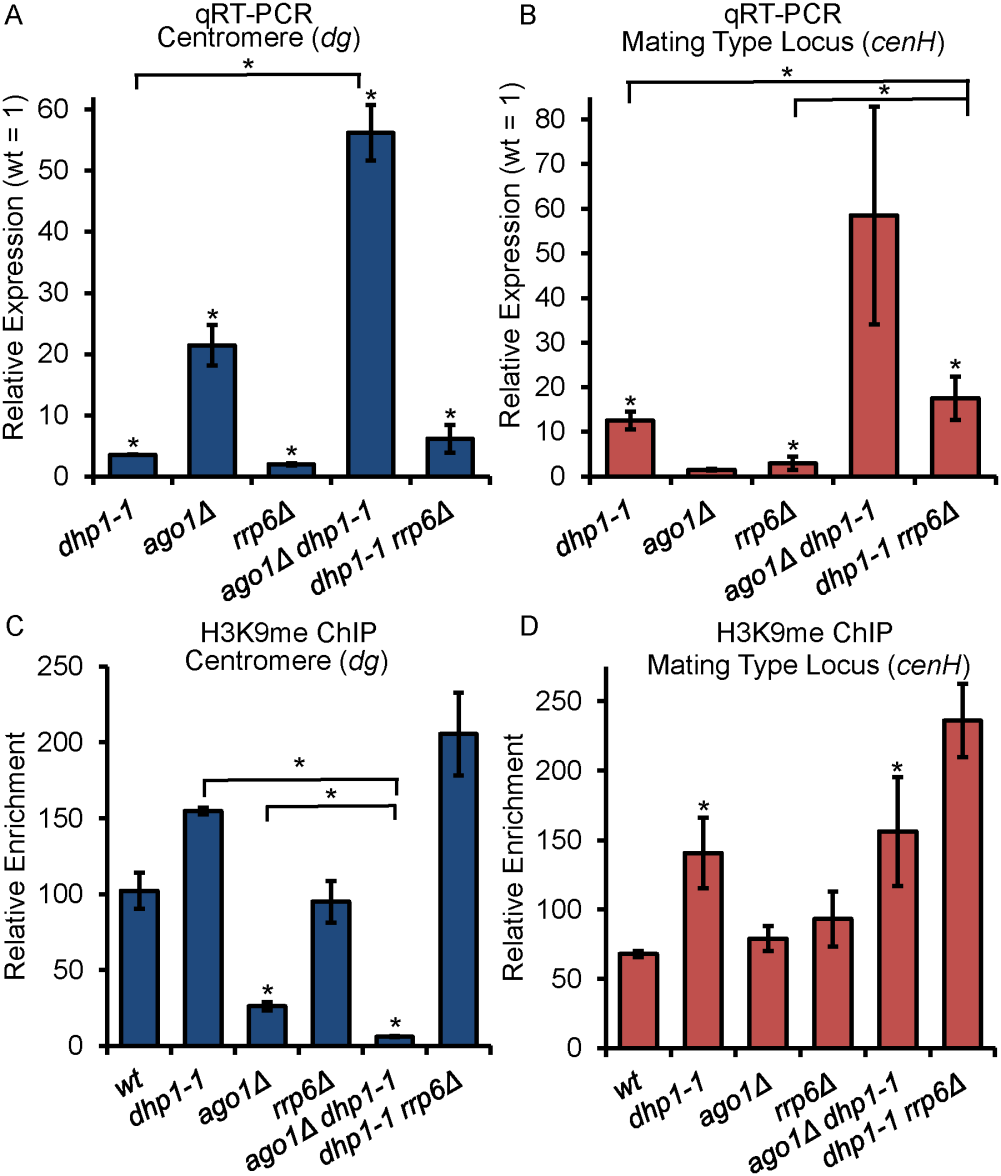
Dhp1-mediated silencing occurs independently of RNAi and the exosome. (A-B) qRT-PCR analysis of silenced repeat regions in the centromere (A) and mating type locus (B) shows negative genetic interactions between *dhp1-1* and *ago1Δ* or *rrp6Δ*. (C-D) H3K9me_2_ ChIPs show the enrichments of H3K9me mark at indicated strains. Relative enrichments of H3K9me_2_ at indicated genomic regions were normalized to *leu1*^*+*^. * *p* ≤ 0.05 as determined by Student’s *t* test comparing the indicated sample values with the wt values for qRT-PCR and qChIP. Significance between the single mutants and double mutants is indicated by horizontal lines linking the compared samples.

We consistently observed a stronger silencing defect in *dhp1-1* at the mating type locus than the pericentromeric region (Figs 1–3). RNAi is known to play a major role in silencing centromeric repeats but only partially contributes to silencing at the mating type locus [64, 65]. These results suggest that Dhp1-mediated silencing might be distinct from that of RNAi. To test this, we combined *dhp1-1* with a deletion of *ago1*, the sole Argonaute protein in *S*. *pombe* [62], and analyzed the silencing defect at the centromeric region and the mating type locus by qRT-PCR. Consistent with previous findings, loss of Ago1 caused an upregulation of centromeric repeat transcripts (Fig 6A) and did not show an obvious silencing defect at the mating type locus (Fig 6B). Whereas *dhp1-1* exhibited a modest increase in transcription at the centromere and the mating type locus, a double mutant *dhp1-1 ago1Δ* showed a large increase beyond the cumulative effects of either single mutation (Fig 6B), indicating that Dhp1 contributes to heterochromatic silencing in a pathway parallel to RNAi. Our conclusion was also supported by the expression data shown in Fig 3B: we consistently observed more relative expression of repeats in *dhp1-1 dcr1Δ* double mutant cells compared to that of *dcr1Δ* or *dhp1-1* single mutant cells from both the centromeric region and mating type locus, further indicating an RNAi-independent role of Dhp1.

Many transcripts degraded by RNAi are also targets of Rrp6 [42], the catalytic subunit of the nuclear exosome required for rapid elimination of cryptic unstable transcripts (CUTs) [73–75]. Its RNA degradation activities act in parallel with RNAi to promote heterochromatin assembly [43, 50]. Since Dhp1 is an exoribonuclease and plays an independent role from RNAi, we next wondered whether Dhp1 has overlapping function with Rrp6 in the silencing of repeat elements. Indeed, qRT-PCR showed that *dhp1-1 rrp6Δ* double mutant cells have stronger silencing defects at both the centromeric region and mating type locus (Fig 6C and 6D), although the effect is less than that observed in *dhp1-1 ago1Δ*.

We next investigated whether the accumulated silencing defects in double mutants of *dhp1* with *ago1Δ* or *rrp6Δ* are resultant from additive deficiencies of H3K9me_2_. ChIP experiments show that, except for *dhp1-1 ago1Δ* at the centromeric repeats, none of the double mutants exhibit further reduction of H3K9me_2_ compared to single mutants (Fig 6C and 6D), suggesting the role of Dhp1 in epigenetic silencing does not rely on H3 K9 methylation at repeat regions. Notably, combining *dhp1-1* and *rrp6Δ* mutations enhances H3K9me both at the centromeric region and the mating type locus (Fig 6C and 6D). A recent study reported that Rrp6 is required for RNAPII termination at specific targets [73]. Our observation of enhanced H3K9me occurring in *dhp1-1 rrp6Δ double* mutant cells suggests that transcription termination defects impair RNAPII transcription and favor the induction of RNA-mediated chromatin modification such as H3K9 methylation. In *dhp1-1 rrp6Δ*, the compounded silencing defect must be overcompensating for the increased silencing effect of the additively enhanced H3K9me (S7 Fig and discussion). Collectively, these results show that the Dhp1-mediated silencing mechanism is independent of both RNAi and the exosome, and is likely downstream of H3K9me.

### The catalytic activity of Dhp1 is required for its role in epigenetic silencing

**Fig 7:**
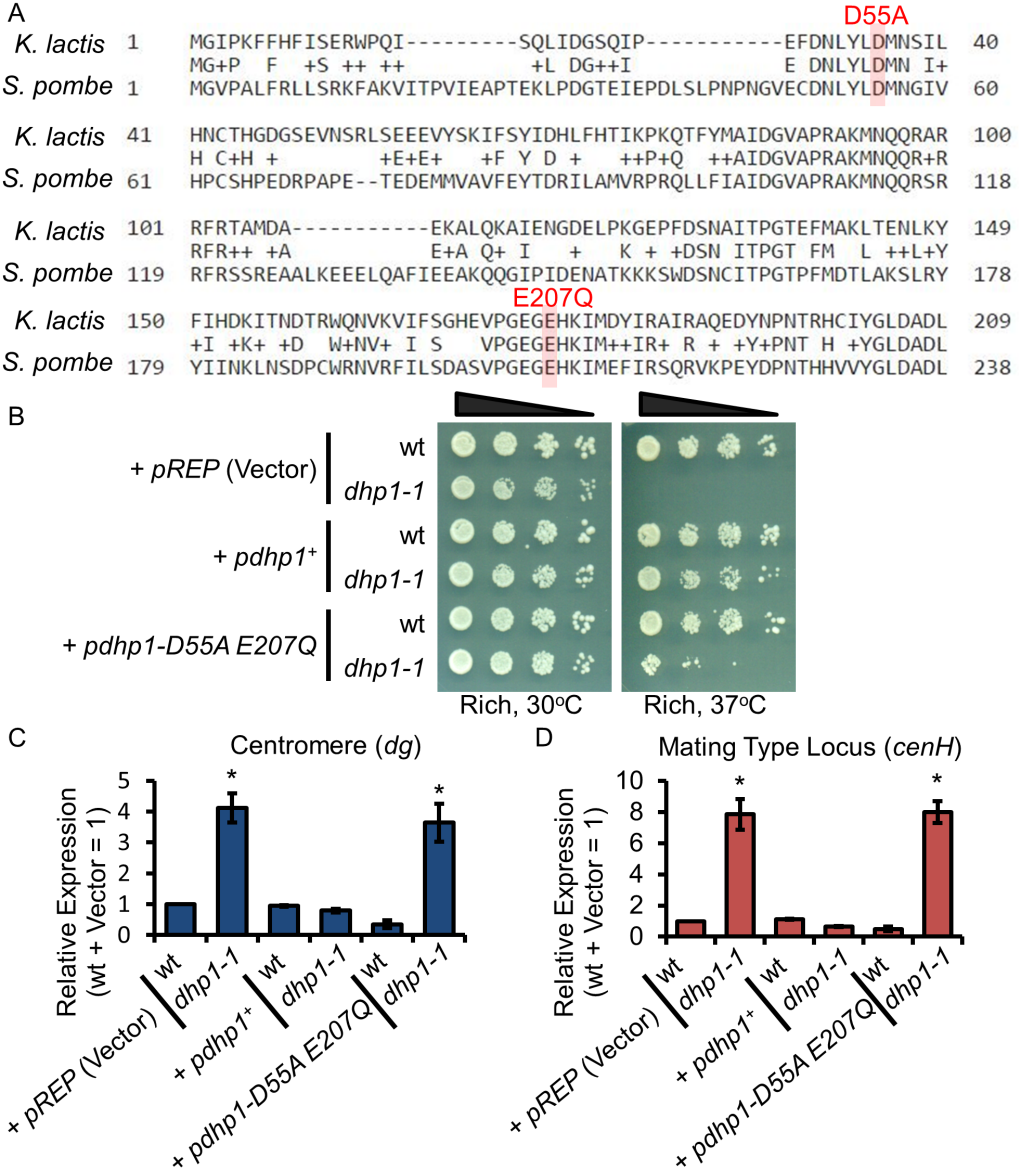
The catalytic activity of Dhp1 is required for silencing. (A) Sequence alignment of the catalytic domains between *K*. *lactis* and *S*. *pombe*. The position of D55 and E207 of Dhp1 in *S*. *pombe* are highlighted. (B) A ten-fold serial dilution assay shows *dhp1-1* cells carrying indicated plasmids grown on standard SC-leucine medium at 30°C or 37°C. (C-D) qRT-PCR showing relative *dg* (C) and *cenH* (D) transcript levels in *dhp1-1* cells carrying indicated plasmids. *P ≤ 0.05 as determined by student’s *t* test comparing the indicated sample values with the WT for qRT-PCR.

Dhp1 is a conserved 5’-3’ exoribonuclease [44, 46]. Previous studies of Xrn1 in *Kluyveromyces lactis* (*K*. *lactis*) indicated that switching the acidic aspartate at position 35 or glutamate at position 178 to neutral residues, such as alanine (D35A) or glutamine (E178Q), completely abolished enzymatic activity [76]. In *S*. *pombe*, Dhp1D55 and E207 are conserved residues corresponding to *K*. *lactis* Xrn1D35 and E178 (Fig 7A). To test whether the RNA processing activity of Dhp1 is important for its role in epigenetic silencing, we generated plasmids carrying a copy of *dhp1* with both D55 and E207 mutated (*dhp1-D55A E207Q)*, which abolishes the catalytic activity of Dhp1. A plasmid carrying a wildtype allele of *dhp1*^*+*^ can rescue both the *ts* phenotype and the silencing defect of *dhp1-1* analyzed by dilution assays (Fig 7B) and qRT-PCR (Fig 7C and 7D), suggesting that the wildtype allele can completely complement the C-terminal truncated form of *dhp1*, further supporting that there is no dominate negative effect of *dhp1-1*. However, the plasmid carrying the catalytic mutant could not rescue the *ts* phenotype (Fig 7B) or the silencing defect of *dhp1-1* at the centromeric region and the mating type locus (Fig 7C and 7D), indicating that the catalytic activity of Dhp1 is essential for its role in epigenetic silencing.

### Dhp1 plays a role in post-transcriptional silencing

In *S*. *pombe*, epigenetic silencing requires cooperation between the TGS and PTGS pathways [15, 62, 64]. As a classic example of PTGS, RNAi allows processing of RNAs transcribed from these regions to facilitate or reinforce heterochromatin assembly in a RNAPII-dependent manner [15, 64]. In this process, siRNAs maintain the feedback loop and propagate heterochromatin. RNAPII activity is required for generating precursors of siRNA and thereby is crucial for heterochromatin assembly [68, 77]. Additionally, RNAPII may have a more direct role in epigenetic silencing because mutation of RNAPII subunits, splicing factors, and RNA processing machineries impair heterochromatin [68, 77–79].

**Fig 8:**
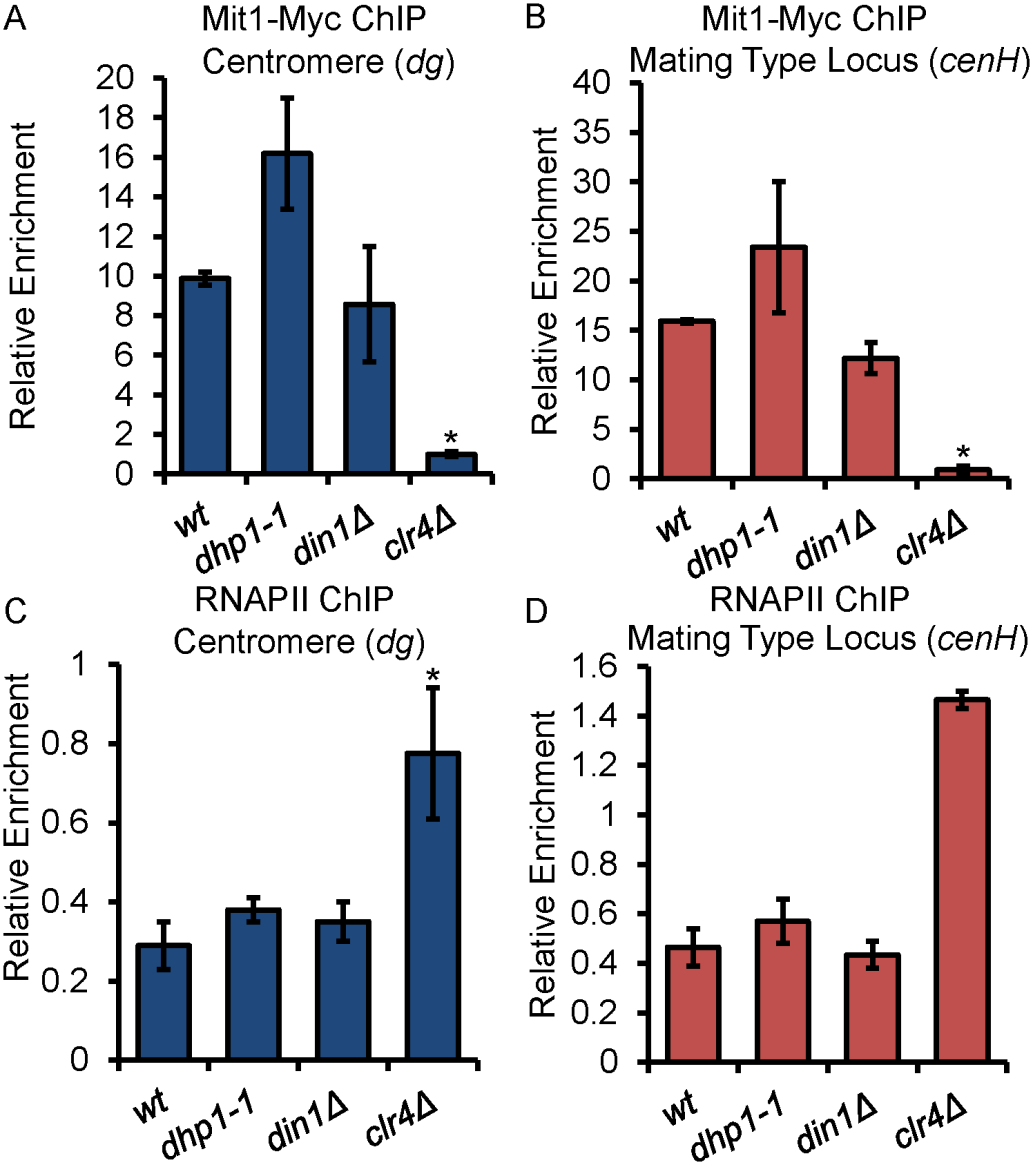
Transcriptional gene silencing (TGS) is not reduced in *dhp1-1*. (A-B) Mit1-Myc ChIPs were carried out using the indicated strains and show that recruitment of SHREC is not decreased in *dhp1-1*. (C-D) RNAPII ChIPs were performed using the indicated strains and reveal no change of RNAPII occupancy in *dhp1* mutant. Relative enrichments of Mit1-Myc (A-B) or RNAPII (C-D) at the centromere (A and C) and the mating type locus (B and D) were normalized to *leu1*^*+*^. * *p* ≤ 0.05 as determined by Student’s *t* test comparing the indicated sample values with the wt values.

TGS relies on heterochromatin which is mediated by histone modifications that recruit silencing effectors [4]. In addition to the H3K9 methyltransferase Clr4 and HP1 family proteins, HDACs are critical mediators of all three phases of heterochromatin formation [80–82]. Especially, deletion of class II HDACs *clr3* or sirtuin *sir2* cause marked reduction of H3K9me across the centromeric regions and mating type locus [80, 82]. To gain further insight into the function of Dhp1 in TGS or PTGS, we compared the localization of Mit1-Myc, one of the core subunits of SHREC (Clr3 complex) at the centromeric regions and the mating type locus in wildtype, *dhp1-1*, *din1Δ*, and *clr4Δ* cells (Fig 8A and 8B). Mit1-Myc is a fully functional allele of Mit1, and has been employed in previous studies [80]. Unlike *clr4Δ*, which abolishes the localization of Mit1, *dhp1-1* does not show any reduction of Mit1 localization at these regions (Fig 8A and 8B), indicating that the localization of SHREC is not reduced. Next, we combined *dhp1-1* with either *clr3Δ* or *sir2Δ*, and examined the silencing of repeat regions in wildtype, single and double mutant cells (S8 Fig). RT-PCRs show that all double mutant cells have enhanced silencing defects compared to single mutants suggesting overlapping functions between Dhp1 and these HDACs (S8 Fig). These results suggest that Dhp1 likely has a major role in PTGS and acts in a distinct pathway parallel to SHREC-mediated TGS.

To further investigate the role of Dhp1 in TGS or PTGS, we analyzed the relationship between Dhp1 and RNAPII in heterochromatin formation. First, we attempted to combine *dhp1-1* with *rpb7-G150D*, which carries a mutation on the RNAPII subunit Rpb7 and has a specific defect in centromeric pre-siRNA transcription [68]. Surprisingly, combined mutation of *dhp1-1* with *rbp7-G150D* is lethal, suggesting the presence of a compensatory mechanism between Dhp1 and RNAPII to ensure proper regulation of the transcriptome. We next combined *dhp1-1* with *rpb2-m203*, a mutant of the second largest subunit of RNAPII [77]. This mutation does not affect the global transcriptional activity of RNAPII [77]. Instead, it specifically influences the generation of siRNA [77]. Our data indicates that Dhp1 plays a role in a pathway parallel to RNAi in the silencing of repetitive regions (Fig 6). Therefore, the Dhp1-mediated silencing defect is unlikely to be linked through *rpb2-m203*. Indeed, *rpb2-m203 dhp1-1* double mutant cells are viable, and have a stronger silencing defect at the centromere region than either single mutant (S9 Fig), indicating independent, parallel functions in epigenetic silencing.

Heterochromatic regions commonly exclude RNAPII as a mechanism of TGS, but PTGS mechanisms occur downstream of RNAPII recruitment. We wondered whether, like RNAi and the exosome, Dhp1 plays a major role in PTGS. Therefore, RNAPII inclusion or exclusion from chromosomal regions will serve as an indicator to elucidate the function of Dhp1 in transcriptional and/or post-transcriptional actions. We mapped RNAPII occupancy in wildtype and *dhp1-1* by ChIP using *clr4Δ* as a control (Fig 8C and 8D). Loss of Clr4 completely abolishes heterochromatin, thereby shows a strong TGS defect as indicated by dramatically increased RNAPII occupancy at the repetitive regions. However, no difference of RNAPII occupancy was observed between *dhp1-1* and the wildtype control at repetitive regions, suggesting the role of Dhp1 is not in TGS, but rather PTGS (Fig 8C and 8D).

**Fig 9:**
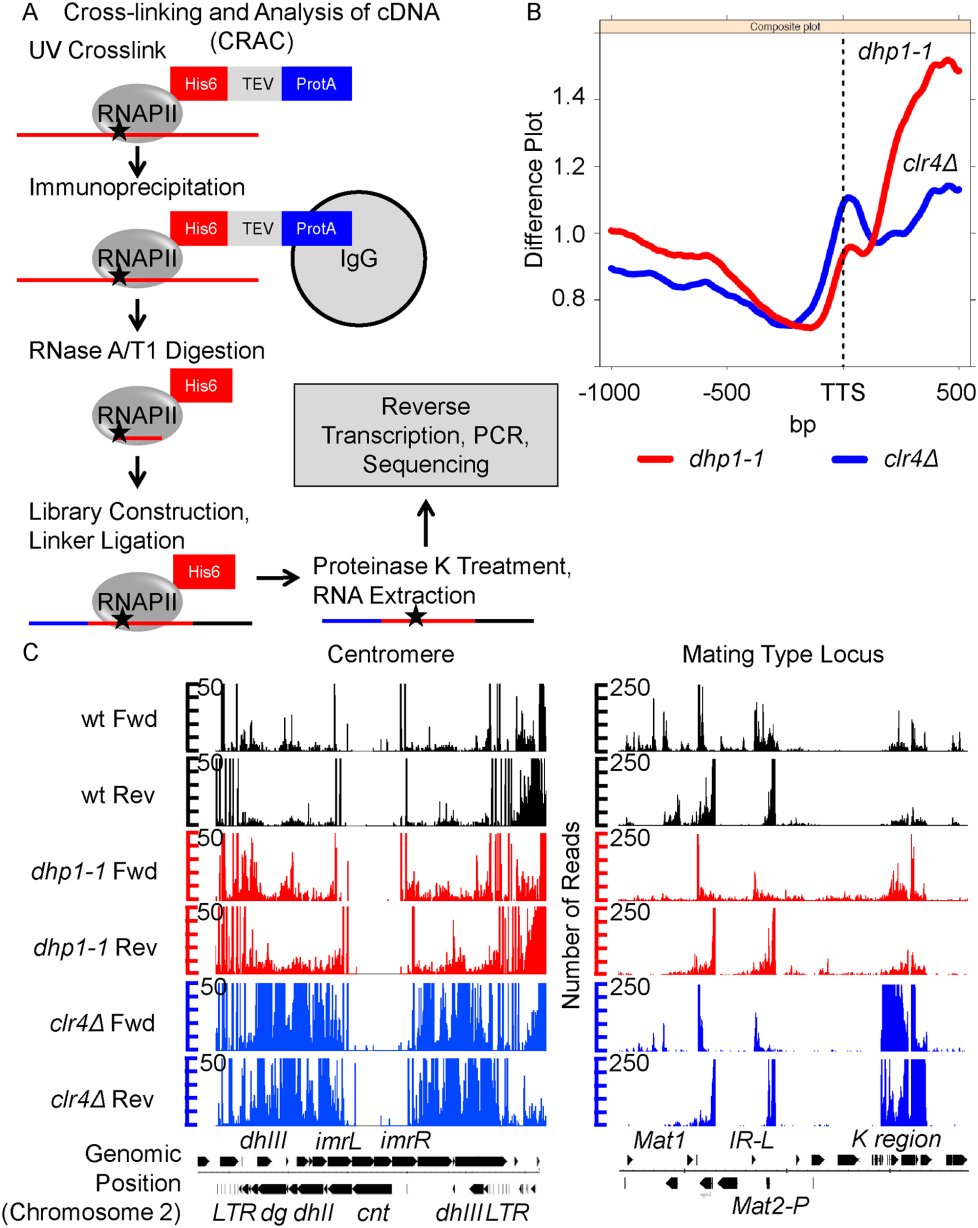
Dhp1 does not affect the binding of RNAPII to repeat transcripts. (A) Cross-linking and analysis of cDNA (CRAC) followed by expression profiling reveals RNAPII targets on a genome-wide scale. (B) CRAC was carried out using a carboxyl-terminal HTP-tagged subunit of RNAPII, Rpb2-HTP. The difference plot maps the relative enrichments (Mutant/wt) of RNAPII substrates averaged across the genome for each mutant in relation to the transcriptional termination site (TTS). RNAPII substrates are relatively enriched after the normal termination site of transcripts in *dhp1-1* due to a transcriptional termination defect. (C) No dramatic changes in enrichment of RNAPII substrates were observed across the centromeric regions and the mating type locus between wt and *dhp1-1* cells, suggesting that Dhp1 does not affect silencing by TGS, but likely through a PTGS mechanism. The chromosome position of the genomic region is displayed below the figure (Fwd, forward strand; Rev, reverse strand).

Decreased transcription termination demonstrated in *dhp1* mutants may reduce the level of available RNAPII complexes for initiation of transcription and could mask the true extent of silencing. Additionally, protracted RNAPII association at a given locus due to stalling might confound ChIP results. To ensure that the true activity of RNAPII was measured, we performed a genome-wide survey of RNAPII targets using Cross-linking and analyses of cDNA (CRAC) in wildtype and *dhp1-1* cells (Fig 9). This assay mapped the genome-wide distribution of RNAPII and also monitored the RNAPII complexes actively synthesizing RNAs [83](Fig 9A). A genome-wide study is necessary in this case as Dhp1 may serve distinct roles in euchromatin and heterochromatin, as genome-wide expression profiling suggested (Fig 1 and S3 Fig). In euchromatic regions, defects in terminating RNAPII transcription caused by the *dhp1* mutation led to an accumulation of unreleased RNAPII complexes at the 3’end of genes in *dhp1-1* (Fig 9B). However, the same phenotype was not observed in *clr4Δ* cells, indicating that loss of *clr4* causes no transcription termination defect. In heterochromatic regions, although *clr4Δ* dramatically enhanced RNAPII-RNA associations at the centromeric region and mating type locus compared to that of wildtype cells due to complete loss of TGS and partial loss of PTGS, such a difference was not detected upon mutation of *dhp1* (Fig 9C). Given the fact that mutation of *dhp1* leads to substantial upregulation of repeat transcripts (Fig 1C) without reduction of H3K9me at repetitive regions (Figs 2 and 6), and only marginally affects RNAPII occupancy and its association with repeat transcripts (Figs 8C, 8D and 9), the results support a primary role of Dhp1 in PTGS.

## Discussion

It is well appreciated that RNA processing pathways, including RNAi and the exosome, play crucial roles in the assembly of heterochromatin and elimination of unwanted transcripts [27]. Here we identify a novel RNA processing mechanism mediated by the essential 5’-3’ exoribonuclease Dhp1, which participates in epigenetic silencing in *S*. *pombe* independent of both RNAi and the exosome. Interestingly, defective gene silencing at two major heterochromatic regions, the centromeric region and the mating type locus, was observed selectively in *dhp1-1* and not in *din1Δ* or *rhn1Δ* cells (Fig 1 and S4 Fig). This result suggests that Dhp1 engages in a silencing pathway that is beyond its Din1- or Rhn1-related activity. Our further genetic analyses support a role for Dhp1-mediated silencing in the sequential establishment of epigenetic silencing (Figs 3–5), parallel to RNAi and the exosome (Fig 6), and most likely involved in PTGS via its RNA processing activity (Figs 7–9).

### RNA processing pathways in epigenetic silencing

**Fig 10:**
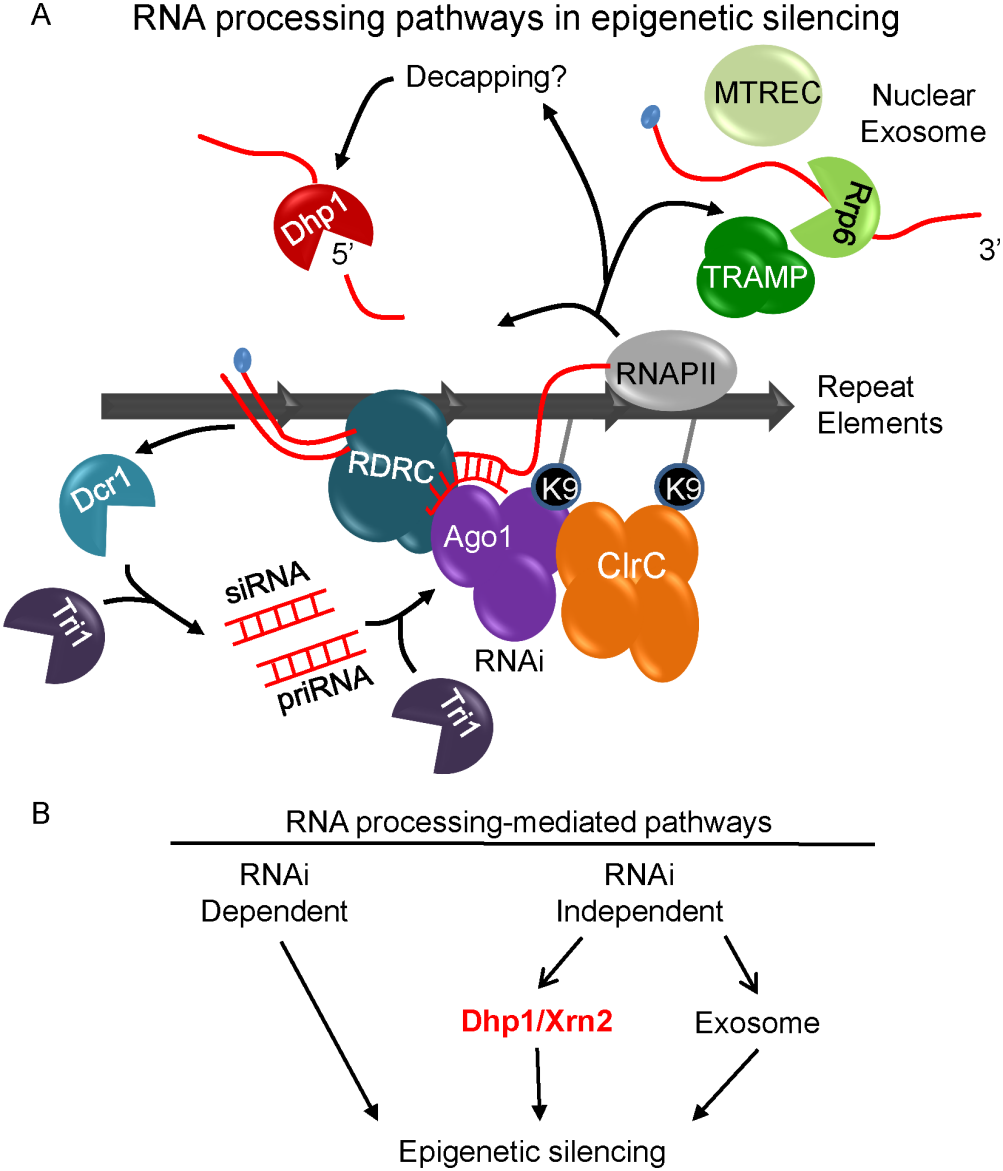
Dhp1 represents a novel RNA processing mechanism which mediates epigenetic silencing independent of both RNAi and the exosome. (A) Aberrant transcripts produced by RNAPII are subject to degradation via three distinct PTGS pathways. RNAi involves the degradation of the transcript by Dcr1 or Triman (Tri1), producing siRNAs. The exosome degrades transcripts in a 3’ to 5’ direction with guidance from its cofactors, the TRAMP and MTREC complexes. Dhp1 mediates a distinct third pathway of post-transcriptional gene silencing via its 5’ to 3’ exoribonuclease activity. Together, these mechanisms function to initiate and maintain the epigenetic silencing of repeat RNAs. (B) RNA processing-mediated mechanisms in epigenetic silencing. We propose that Dhp1/Xrn2-mediated silencing is a novel RNAi- and exosome-independent processing mechanism in gene silencing.

In spite of the crucial role for RNAi in heterochromatin assembly, heterochromatin is not completely abolished in RNAi mutants indicating that other pathways are involved [50, 65, 84]. These pathways are mediated by DNA-binding factors, RNA or RNAi-independent RNA processing factors [50, 61]. In *Arabidopsis*, the flowering repressor gene FLC is thought to provide links between RNA processing activities and chromatin regulation in gene silencing [85]. In *S*. *pombe*, recent studies reveal the nuclear exosome, which governs RNA quality control and ensures the elimination of unwanted RNAs, exists as an RNAi-independent silencing mechanism [42, 43, 50, 61]. Co-activators of the exosome, including TRAMP and MTREC, which help to recognize and degrade its substrates, are also connected to epigenetic silencing without affecting H3K9me, thereby play a major role in PTGS [61, 86]. Additional studies on Triman, a 3’-5’ exonuclease in *S*. *pombe*, show that it generates Dicer-independent primal RNAs and is required for initiation of heterochromatin assembly via a mechanism requiring Ago1 [87]. In this study, we described a novel pathway involving Dhp1, a conserved RNA 5’ to 3’ processing enzyme that contributes to PTGS (Fig 10A). We propose that three RNA processing activities, RNAi, the exosome, and Dhp1/Xrn2 degrade repetitive transcripts to mediate the post-transcriptional gene silencing of repeat transcripts (Fig 10B).

### The role of Dhp1 in initiation, spreading, and maintenance of epigenetic silencing

Heterochromatin assembly is a dynamic process with distinct steps [4]. It is nucleated at genomic regions containing highly repetitive DNA elements and spread to surrounding regions [88]. Its structure is recaptured during DNA replication and maintained through cell division [14]. Silencing factors often participate at discrete step(s) rather than throughout the process. In particular, RNA-mediated silencing pathways are often required to nucleate heterochromatin formation [9]. Once silencing is established, these factors are dispensable [13]; the heterochromatic state persists in the absence of the initial stimulus. For example, at the mating type locus of *S*. *pombe*, RNAi machinery cooperates to nucleate heterochromatin assembly but is dispensable for its inheritance [13]. The re-establishment assay clearly indicates that Dhp1 is indispensable for efficient establishment of silencing at heterochromatic repeat regions (Fig 3). RNAi is well-known as the major nucleation pathway at centromeric regions but not at the mating type locus [64, 65]. Interestingly, *dhp1-1 ago1Δ* double mutant cells have cumulative defects at the mating type locus indicating separate functions of these two pathways (Fig 6). Unlike Triman, which requires Argonaute to be loaded on longer RNA precursors [87], Dhp1 has an Argonaute-independent role, although we cannot rule out the possibility that the slicer activity of Ago1 may also contribute to the generation of the substrates for Dhp1. Since RNAi itself can initiate heterochromatin formation, we observed re-establishment of heterochromatin at repetitive elements in *dhp1-1* cells following *clr4*^*+*^ complementation, although the restoration was not complete (Fig 3). These results suggest that Dhp1 plays a unique but overlapping role in heterochromatin nucleation in concert with RNAi. It is possible that the Dhp1-mediated degradation of heterochromatic repeat transcripts is required for *de novo* assembly of heterochromatin through recruiting silencing effectors, similar to RITS [36, 38, 39]. It is also possible that the processing activity of Dhp1 is involved in generating the primary small RNAs that contribute to initiation of epigenetic silencing as suggested for the role of the exosome in heterochromatin assembly [61].

In addition to defective nucleation, the H3K9me mark in *dhp1* mutants is reduced in the reporter genes embedded at the repetitive regions, suggesting a spreading defect (Fig 2B and 2C). The assay analyzing the spreading of H3K9me from an ectopic nucleation center to the surrounding regions indicates that Dhp1 facilitates the spreading of the heterochromatic mark (Fig 4). Although it is unclear how transcription-mediated spreading of heterochromatin occurs, it is possible that impaired transcription termination in the *dhp1* mutant affects the rate of histone turnover during transcription and thereby impedes the spreading of H3K9me.

In addition to its role in initiation and spreading, we provide evidence to show that Dhp1 functions in the maintenance of pre-established silencing (Fig 5). How does Dhp1 function in the maintenance of silencing? It is known that heterochromatin maintenance relies on the binding of Swi6 and Clr4 to methylated H3K9, which facilitates recapitulation of the specific chromatin configuration following DNA replication [38, 70]. In addition, Swi6 and HP1 proteins work as binding platforms, recruiting other histone modifiers and with the factors that are involved in replication-coupled heterochromatin assembly, such as chromatin assembly factor 1(Caf1) [65, 80, 89, 90]. Although the levels of H3K9me_2_ at the repeat regions are not decreased upon mutation of *dhp1*, the dynamic binding of Swi6 could still be affected. In addition to H3K9me, Swi6 is also reported to bind “repellent” RNAs that antagonize the heterochromatic silencing [91]. Thus, Dhp1-mediated elimination of RNA may facilitate the dynamic binding of Swi6 to heterochromatin, and thereby ensure the maintenance of the silenced chromatic domains.

### Silencing effects differ between the centromeric regions and the mating type locus in the *dhp1* mutant

Although the centromeric regions and the mating type locus are assembled by heterochromatin, they occupy different chromosomal contexts and use distinct strategies to target heterochromatin [4]. Notably, the effects in double mutants of *dhp1* with *ago1Δ* or *rrp6Δ* are different at centromeres and the mating type region (Fig 6). At the centromeric region, RNAi is the major pathway [64]. Therefore, as expected, the *ago1Δ* single mutant exhibits a severe silencing defect and decreased H3K9me mark (Fig 6 and S7 Fig). Compared to the already radically impaired silencing phenotype in the *ago1Δ* single mutant, the *dhp1-1 ago1Δ* double mutant shows even higher levels of repeat transcripts and lower levels of H3K9me_2_ (Fig 6), suggesting that transcripts produced from repeat regions in RNAi-deficient cells, are likely targets of Dhp1. In contrast, without Rrp6/exosome, RNAi machinery is still functional. Therefore, we only observe a moderate silencing defect at the centromeric region in the *dhp1-1 rrp6Δ* double mutant (Fig 6). At the mating typing locus, at least three pathways initiate heterochromatin assembly and target H3K9me [4, 27]. It is not surprising that the *dhp1-1 ago1Δ* double mutant maintains a high level of H3K9me_2_ (Fig 6); other pathways may compensate for loss of function for both Dhp1 and RNAi at the mating type locus [4]. In addition, transcription termination defects caused by *rrp6* and *dhp1* mutation may contribute to the increased level of H3K9me seen at both centromere and mating type locus (S7 Fig). Interestingly, cells containing the *dhp1* mutation consistently show a stronger silencing defect at the mating type locus even in the presence of higher levels of H3K9me (Figs 1–3 and 6 and S7 Fig), suggesting that Dhp1-mediated silencing occurs primarily downstream of H3K9me, likely as a mechanism of PTGS.

### The function of Dhp1 in PTGS

In *S*. *pombe*, TGS and PTGS are intertwined. In TGS, heterochromatin greatly limits the access of RNAPII, allowing only a low level of transcription from highly repetitive DNA regions. RNAs transcribed from these regions are subject to PTGS by RNAi machinery, in which they are processed into siRNAs in order to feedback on chromatin to facilitate the assembly and propagation of heterochromatin [27, 88]. The silencing defect in *dhp1-1* is unexpected considering that compromised transcription termination would weaken RNAPII transcription and delay the release of RNA from the site of transcription, which may then enhance the assembly of heterochromatin mediated by RNA as suggested by previous studies [48, 50]. To elucidate whether Dhp1 plays a major role in TGS, we used ChIP analysis to map H3K9me and SHREC (Figs 2, 8A and 8B), which have well-studied functions in TGS at repeat regions. If Dhp1 plays a role in TGS, we would expect to observe reduced enrichment of H3K9me and SHREC at the endogenous repetitive regions in *dhp1-1*. No reduction of enrichment occurred however for either H3K9me_2_ or SHREC at endogenous repetitive regions in *dhp1* mutants, suggesting that the role of Dhp1 in gene silencing is primarily associated with PTGS rather than TGS (Figs 2, 8A and 8B). We further investigated RNAPII occupancy and the levels of actively transcribing RNAPII at repeat regions in wildtype and mutant cells using ChIP and CRAC respectively (Figs 8C, 8D and 9). A role in TGS for Dhp1 would be suggested by increased RNAPII occupancy occurring at repetitive regions in *dhp1-1*, as RNAPII in the context of impaired TGS would associate more frequently with heterochromatic transcripts. In contrast, no increase would implicate a role for Dhp1 in PTGS. Our RNAPII ChIP results clearly show no difference between *dhp1-1* and wildtype, implicating a PTGS role for Dhp1 (Figs 8C and 8D). Additionally, we showed that the catalytic activity of Dhp1 is required for its role in epigenetic silencing, providing strong evidence to support that the RNA processing role of Dhp1 is associated with PTGS (Fig 7). Although our results pinpoint the primary role of Dhp1 in epigenetic silencing through PTGS, completely discounting a function of Dhp1 in TGS is a challenge as Dhp1/Rat1/Xrn2 has well-established activity that is linked to RNAPII.

### RNAPII-associated function of Dhp1

RNAPII transcription and its associated activities are required for heterochromatin assembly. As a result, loss of silencing was reported to correlate with defective RNAPII transcription [68, 77, 92–94]. Is the RNAPII-linked function of Dhp1 related to epigenetic silencing? In agreement with reported termination defects upon mutation of Dhp1 and Din1, our expression profiling showed accumulation of 3’ untranslated transcripts at many genes in these mutants (S3 Fig). To execute its function in RNAPII transcription termination, Dhp1/Rat1 exonucleases target the downstream fragments produced by cleavage at the polyA site during 3’ end processing [44–46]. The processed mRNAs are packed into nuclear RNA transporting cargos and exported to the cytoplasm for translation. Since this action of Dhp1/Rat1 in transcription termination lies downstream of mRNA processing and packaging, defects of Dhp1/Rat1 are unlikely to dramatically influence the amount and the quality of coding mRNAs. Indeed, at least at the permissive temperature, we did not observe significant alterations of coding transcripts in the *dhp1* mutant (S2 Table). Rather, the remarkable differences observed in the transcriptome were seen at non-coding regions (S3 Fig). Recently, Rat1 in budding yeast was reported to maintain the balance of RNAPII CTD phosphorylation, and therefore plays a role in transcription elongation [95]. This finding suggests that Rat1 may have more complex roles in transcription than previously thought. In addition, neither loss of Rnh1 nor Din1 causes growth defects or silencing defects as seen in the *dhp1* mutant (Figs 1 and 2 and S4 Fig), raising the question about which role of Dhp1, transcription or RNA quality control, is essential for cell growth and silencing. In this study, we indeed observed higher enrichment of H3K9me_2_ at the endogenous repetitive regions in the *dhp1* mutant (Figs 2 and 6). This observation is in agreement with a study showing that an impaired Paf1 complex is sufficient to induce RNAi-mediated epigenetic silencing *in trans* at euchromatic loci, likely through its termination defect [48]. While a parallel study reported significant reductions of H3K9me_2_ in *dhp1* mutants at all major heterochromatic regions [59], we only observed reduced H3K9me at reporter genes but not at the repeat regions. It is likely that the discrepancies in the H3K9 methylation data are due to differences in culturing conditions. To minimize the pleiotropic impacts caused by *dhp1* mutation and avoid the antagonistic effect of high temperature (37°C) for heterochromatin formation [96], we collected data at a permissive condition (30°C) without shifting cell cultures to 37°C, the restrictive condition applied in the parallel study [59]. Therefore, our results are more likely to accurately represent the true effect of Dhp1 in epigenetic silencing. In addition, we provided evidence to show that Dhp1-mediated silencing is independent of RNAi (Fig 6). Overall, the role of Dhp1 in epigenetic silencing at major heterochromatic regions cannot be explained by its known function in transcriptional termination. It is possible that RNAPII may couple repeat transcription with its degradation by Dhp1. A second possibility is that RNAPII may help “discriminate” noncoding pericentromeric repeat RNAs from general pre-mRNAs so that the former can be degraded by Dhp1. The basis for this selection may be the aberrant (double-stranded or abnormally capped) structure of the transcribed RNA. Alternatively, the chromatin structure of the transcribed repeat region may somehow determine the fate of the transcripts, feeding into RNAi-, exosome-, or Dhp1-mediated silencing.

### Enzymatic activity of Dhp1 in epigenetic silencing

The catalytic activity of Dhp1 is required for its role in epigenetic silencing (Fig 7). By what mechanism are the substrates for Dhp1-mediated silencing produced? Due to the strong conservation of the active site, it is likely that the mechanisms of Xrns are very similar [97]. The crystal structure of *Drosophila* XRN1 indicates that substrates are limited to 5’ monophosphate RNAs because larger structures, such as m7G Cap or triphosphorylated RNAs, do not fit into the pocket [98]. Hence, the RNA pyrophosphohydrolase activity of Din1 seems necessary for the generation of monophosphorylated RNA substrates for Dhp1, especially for decapping and RNA quality control. However, only Dhp1, not Din1, is essential for viability and epigenetic silencing. In addition, unlike Dhp1, Din1 and its orthologs are not widely conserved [47, 99]. Since Din1 is not essential and is not necessary for epigenetic silencing, an endoribonuclease or an extra RNA pyrophosphohydrolase likely produces the substrates for Dhp1 in silencing.

In yeast, abnormal pre-mRNAs are degraded rapidly from both 5’ and 3’ ends by Rat1/Xrn2 and the nuclear exosome, respectively, with the exosome playing a dominant role [100]. In human cells, XRN2 appears to be more crucial for degradation of abnormal pre-mRNAs than the exosome [101]. Given the fact that Xrn2 is conserved from yeast to humans, our results may yield insights broadly applicable to the gene silencing field, including mammals. Dhp1/Xrn2 may represent a more generalized mechanism of an RNA-based form of silencing. Future studies identifying additional Dhp1/Xrn2 interacting proteins may help to address these questions.

## Materials and Methods

### Yeast strains, cell culture

*S*. *pombe* strains used in this study are listed in S1 Table. Cells were cultured using standard procedures for growth and manipulation [102]. Epitope-tagged and deletion mutant strains were engineered using standard PCR methods as described previously [103]. Double mutants were constructed via genetic crossing followed by tetrad dissection. For dilution assay, liquid cultures were diluted in series (1:10) and plated using a pin transfer tool on YEA media (Rich, N/S), low adenine YE media, or YEA media containing either 20 μg/ml TBZ or 850 μg/ml 5-FoA. All cultures were grown at 30°C (or 37°C where indicated). The strains used for cross-linking and analyses of cDNA (CRAC) carry a carboxyl-terminal HTP-tagged subunit of RNAPII, Rpb2-HTP. An HTP tag contains a 6X- His epitope and a protein A epitope separated by a Tobacco Etch Virus (TEV) protease cleavage site [83]. Strains carrying either *KΔ*∷*ade6*^*+*^off or *KΔ*∷*ade6*^*+*^on were isolated and saved as previously described [71].

### Plasmid construction

To generate p*dhp1*^*+*^, a PCR fragment amplified using oligos Dhp1-BamHI-Fw and Dhp1-Pst1-RV contains a wildtype *dhp1*^*+*^ gene including promoter, open reading frame, and 5’ and 3’ untranslated regions. The PCR fragment was digested by BamHI and PstI and ligated into a pREP41 digested with the same restriction enzymes (BamHI/PstI). After BamHI/PstI digestion, pREP41 lost its nmt promoter. The resulting p*dhp1*^*+*^ expresses the wildtype *dhp1*^*+*^ driven by its endogenous promoter. p*dhp1D55A E207Q* was generated using a QuickChange Site-Directed mutagenesis kit (Stratagene) based on p*dhp1*^*+*^. p*clr4*^*+*^ is a plasmid carrying a DNA fragment containing a wildtype *clr4*^*+*^ driven by its endogenous promoter as previously described [50].

### Strand specific qRT-PCR

Total RNA was prepared using the MasterPure Yeast RNA Purification Kit (Epicentre). First-strand cDNA was produced with M-MLV Reverse Transcriptase (Promega) using site-specific primers following manufacturer protocols. Real-time PCR was performed on a 7500 Fast Real-Time PCR System (Applied Biosystems) with SYBR Select Master Mix (Applied Biosystems). First-strand cDNA synthesis without reverse transcriptase was performed for negative controls. At least two biological repeats were performed for all experiments. Statistical analysis was performed using a student’s *t* test (two-tailed distribution). Error bars represent standard error of mean (s.e.m). Primers are listed in the S4 Table.

### Fluorescence-based analysis

Mating-type switching-competent (*h*^*90*^) mid-log phase cells (wildtype, *dhp1-1*, or *din1Δ*) were plated on solid sporulation medium (SPA). Cells grew at 30°C for 6 hr, then switched to 37°C for 2 hr, and finally finished the sporulation at 30°C for 12hr. Cells were washed 3X with water. Ten microliter cells in water were spread on a glass slide, and fixed by heat at 70°C. The slides were then covered by 5μl of mounting buffer with DAPI (VECTOR, H1500) and 13mm coverslips. The stained cells were imaged by a confocal microscope.

### Expression arrays

Sample preparation for the expression array and array design were reported previously [104]. The expression profiling is performed as previously described [105]. The composite plot was generated using *GenomicRanges* R-package (version 1.20.5), from the high-resolution part of the microarray (2320 genes). The genes were aligned at the transcriptional termination site TTS (*S*. *pombe* 2007_April annotation) and the geometric means of the ratios (Mutant/wt) were plotted.

### Tandem affinity purification (TAP) and mass spectrometry analysis

Flag-TEV-protein A (FTP)-tagged purification and mass spectrometry were performed as previously described [105].

### Chromatin immunoprecipitation

ChIP experiments were performed as described previously using antibodies against histone H3 (di-methyl K9) (Abcam,Ab1220), RNAPII (Abcam, Hab5408), or Myc (Santa Cruz, A-14) [106]. Real-time PCR was performed on a 7500 Fast Real-time PCR System (Applied Biosystems) with SYBR Select Master Mix (Applied Biosystems). At least two biological repeats were performed for all ChIP experiments. Statistical analysis was performed using a Student’s *t* test (two-tailed distribution). Error bars represent s.e.m.

### Cross-linking and analyses of cDNA (CRAC)

*In vivo* CRAC was performed as described with modifications [83]. Two-liter yeast cultures were grown to an OD_600_≈2 at 30°C. Cells were harvested by centrifugation and cell pellets were resuspended in 2.5L Phosphate-Buffered Saline (PBS) followed by UV-irradiation in a “Megatron” UV-cross-linker (254 nm) for 3 min before cells were pelleted and frozen in liquid nitrogen. The pellets were then lysed by grinding in liquid nitrogen (Resch, MM400) and resuspended in 10 ml of 1x TN150 lysis buffer (10x TN150: 0.5 M Tris-HCl (pH 7.8), 1.5 M NaCl, 1% NP-40). Extracts were clarified by centrifugation (10 min at 4000 rpm and 45 min at 15,000 rpm at 4°C) and incubated with 150 μl of equilibrated IgG Sepharose beads (GE Healthcare) for 1h at 4°C. After two washes with TN1000 buffer (100 mM Tris-HCl (pH 7.8), 2 M NaCl, 0.2% NP-40) and two washes with TN150 lysis buffer, the beads were incubated with GST-TEV protease for 2h at 16°C. The TEV eluates were collected by centrifugation and incubated with 10U of Turbo DNase (Ambion) for 8 min at 37°C followed by incubation with RNase Cocktail Enzyme Mix (Ambion; 0.005 U RnaseA, 0.2 U Rnase T1) for 2 min at 37°C. Guanidine-HCl (0.4g) was dissolved in 500 μl of TEV eluates. NaCl and Imidazole were added to final concentrations of 300 mM and 10 mM, respectively. Samples were incubated with 50 μl of nickel agarose beads (Macherey-Nagel) over night at 4°C. All washes, alkaline phosphatase treatment and 3’ linker ligation were carried out as described except that 40U T4 RNA ligase 2 truncated K227Q (NEB) was used instead of T4 RNA ligase. The beads were incubated in 80 μl phosphorylation mix (16 μl 5x PNK buffer (250 mM Tris-HCl (pH 7.8), 50 mM MgCl_2_, 50 mM β-mercaptoethanol), 200 mM ATP (Sigma, A6559), 20U T4 polynucleotide kinase (NEB), 80U RNase Inhibitor) for 40 min at 37°C. For the ligation of the 5’ linker the beads were resuspended in 80 μl of 5’ ligation mix (16 μl 5x PNK buffer, 80U RNasin, 40U T4 RNA ligase, 100 pmol 5’linker, and 80 mM ATP) and incubated at 16°C. After two washes with wash buffer II (50 mM Tris-HCl (pH 7.8), 50 mM NaCl, 10 mM Imidazole, 0.1% NP-40) the material was eluted with elution buffer (10 mM Tris (pH 7.8), 50 mM NaCl, 150 mM Imidazole and 0.1% NP-40). The final eluate was incubated with 2 M EDTA, 20 μl 20% SDS and 100 μg proteinase K (Ambion AM2548) for 2 hours at 50°C and the RNA was extracted using Phenol-Chloroform followed by ethanol precipitation. Reverse transcription with SuperScript III was performed following the manufacturer’s instructions (Invitrogen) followed by RNase H (NEB) digest (10U) for 30 min at 37°C. The cDNA was amplified and the PCR-product was purified with the Agencourt AMPure XP PCR purification beads (Beckman Coulter) following the manufacturer’s instructions. The quality of the library was verified with the Bioanalyzer 2100 (Agilent) and the Agilent High Sensitivity DNA Kit (Agilent). The amplified library was subject to high-throughput sequencing at BGI-Hong Kong Co. Ltd. The datasets were mapped to the mating type locus of *S*. *pombe* (41249 bp from chromosome 2) and to the full genome of *S*. *pombe* (ASM294v1.17) using Tophat2 software. (Tophat-2.0.14; [107]). Downstream data analysis was performed using R Bioconductor packages.

The mean coverage over mRNA loci was normalized to 20 in the datasets. The difference plot was generated from all protein coding ORFs (5115 genes), aligning them at the annotated transcriptional termination site (TTS) (*S*. *pombe* EF2 annotation). The plot is showing the ratio between the normalized Mutant/wt coverage. Two biological duplicates have been performed for genome-wide analysis for wildtype and *dhp1-1* strains. All microarray and CRAC data sets are available at NCBI GSE77291, GSE77289 and GSE77290.

## Supporting Information

S1 Table List of strains used in this study. (PDF)

S2 Table Relative expression of silencing factors. (PDF)

S3 Table Dhp1-FTP- and Din1-FTP-interacting proteins identified by LC-MS/MS.* (PDF)

S4 Table List of oligonucleotides used in this study. (PDF)

S1 Fig *dhp1* mutant alleles show temperature sensitivity and silencing defects. (A) Ten-fold serial dilutions demonstrate the expression of *otr*∷*ura4*^*+*^ (on 5-FoA-containing media) and the degree of centromeric heterochromatin functionality (on TBZ-containing media). (B-C) *dhp1-1* has stronger silencing defect than *dhp1-2* as evaluated by qRT-PCR at the centromeric region (B) and the mating type locus (C). * *P* ≤ 0.05 as determined by student’s *t* test comparing the indicated sample values with wt values. Error bar represent s.e.m.
(TIF)

S2 Fig *dhp1* mutant alleles are not dominant negative. (A) *dhp1-1 ts* phenotype is rescued by a wildtype *dhp1*^*+*^ allele borne from a plasmid, p*dhp1*^+^. Two independent colonies for *dhp1-1* or *dhp1-2* carrying p*dhp1*^+^ are shown. All cells were cultured on SC-leucine medium. (B-C) Ten-fold serial dilutions show the growth of diploid wildtype (wt), *dhp1-1* (B) or *dhp1-2* (C) heterozygotes and homozygotes on rich medium at indicated temperatures. The expression of *otr*∷*ura4*^*+*^ in *dhp1-2* is assayed on 5-FoA-containing counter-selective media at 30°C (C).
(TIF)

S3 Fig *dhp1-1* and *din1Δ* are defective in RNAPII transcription termination. Expression profiling shows average RNA expression around transcriptional termination site (TTS), normalized to wildtype (Enrichment (log_2_)). *dhp1-1* and *din1Δ* show increased RNA levels after the normal termination site, indicating a transcription termination defect in both although the defect is greater in *dhp1-1*.
(TIF)

S4 Fig *rhn1* mutants do not show a major silencing defect, as seen in *dhp1-1*. (A) Ten-fold serial dilution on plates containing indicated media and cultured at indicated temperature. (B-C) qRT-PCR demonstrates the silencing defect observed in *dhp1-1*. Only minor silencing defects can be detected in *rhn1Δ* by dilution assay, but no silencing defect is observed by qRT-PCR at the (B) centromere or (C) mating type locus. Error bar represent s.e.m.
(TIF)

S5 Fig Meiotic chromosome segregation is defective in the *dhp1-1* mutant. (A) Aberrant meiotic segregation as visualized by DAPI staining. Class I represents normal meiotic segregation, in which each spore from a tetrad receives a similar amount of DNA (blue dots). Both class II and class III represent mis-segregation events during meiotic division resulting in either ≤ 3 dots (class II) or ≥ 5 dots (class III). (B) The percentage of frequency with which the respective phenotypic classes were calculated. More than 150 randomly selected tetrads of each indicated strain were scored.
(TIF)

S6 Fig The purification of Dhp1- and Din1-associated proteins. Brilliant blue stained 4–12% NuPAGE gel showing two-step affinity purification results for Dhp1-FTP and Din1-FTP. The bands shown here represent proteins which have direct physical interaction with the bait proteins, Dhp1-FTP or Din1-FTP, as purifications were carried out in the presence of Benzonase. (WT, untagged control strain; FTP, a Flag-Protein A tandem epitope tag). A list of peptides identified in the purified fractions by LC-MS/MS can be found in S3 Table.
(TIF)

S7 Fig A schematic model for the result shown in Fig 6. Blue boxes represent the effects of combined mutations at the centromeric region; red boxes represent these effects at the mating type locus; and purple boxes represent the overall effects at both loci. Red arrows depict the relative changes in H3K9me and silencing levels compared to wildtype. We propose that the expression of repeat transcripts and the level of H3K9me observed in *dhp1-1* together with either *ago1Δ* or *rrp6Δ* are additive effects of impaired transcription termination and defective RNA degradation (PTGS).
(TIF)

S8 Fig Dhp1 functions parallel to HDACs Clr3 and Sir2 to mediate silencing. qRT-PCR analysis shows negative genetic interactions between *dhp1-1* and *clr3Δ* (A-B) and *sir2Δ* (C-D) for the silencing of centromeric (A and C) and mating type locus repeats (B and D). *P ≤ 0.05 as determined by student’s *t* test comparing the indicated sample values with the wt. Significance between single mutants and double mutants are indicated by horizontal lines linking the compared samples together.
(TIF)

S9 Fig Dhp1-mediated silencing occurs independently of Rpb2. qRT-PCR analysis of silenced repeat regions in the (A) centromere and (B) mating type locus shows a negative genetic interaction between *dhp1-1* and *rpb2-m203*. *P ≤ 0.05 as determined by student’s *t* test comparing the indicated sample values with the wt. Significance between single mutants and double mutants are indicated by horizontal lines linking the compared samples together.
(TIF)
