# Supplementary material for: A Novel Epigenetic Silencing Pathway Involving the Highly Conserved 5’-3’ Exoribonuclease Dhp1/Rat1/Xrn2 in Schizosaccharomyces pombe
Source: PLoS Genet. 2016 Feb 18;12(2):e1005873. doi: 10.1371/journal.pgen.1005873 (PMC4758730; doi:10.1371/journal.pgen.1005873)
Supplement: S1 Table — (PDF) [file pgen.1005873.s001.pdf]

S1 Table: List of strains used in this study (1 of 3)

Strains	*mat*	*leu1*	*ade6*	*his2*	*ura4*	Epigenetic Reporter	Mutation
SPKZ8	*Mat1 Msmt0*	*leu1-32*	*ade6-216*	*his2*	*ura4 DS/E*	*otr1R(Sph1)::ura4⁺*	-
SPFY648	*h⁺*	*leu1-32*	*ade6-210*	*his2*	*ura4 DS/E*	*otr1R(Sph1)::ura4⁺*	-
SPWF48	*Mat1 Msmt0*	*leu1-32*	*ade6 DN/N*	*plus*	*ura4 DS/E*	*otr1R(Sph1)::ura4⁺, mat2::ade6⁺*	-
SPKZ91a	*Mat1 Msmt0*	*leu1⁺*	*ade6 DN/N*	*plus*	*ura4 DS/E*	*otr1R(Sph1)::ura4⁺, mat2::ade6⁺*	-
SPAP259	*Mat1 Msmt0*	*leu1-32*	*ade6-210*	*plus*	-	*ura4::[CenH3.6KB+ade6⁺]*	-
P20	*h⁺*	*leu1-32*	*ade6-216*	*plus*	*ura4 DS/E*	*otr1R(Sph1)::ura4⁺*	-
P1413	*Mat1 Msmt0*	*leu1-32*	*ade6⁺*	*plus*	*ura4-D18*	-	*rpb2-flag-HTpA*
SPJT174(D)	*Mat1 Msmt0*	*leu1-32*	*ade6 DN/N*	*his2*	-	*kΔ::ade6⁺ (off)*	-
SPWF28	*Mat1 Msmt0*	*leu1-32*	*ade6-210*	*his2*	*ura4 DS/E*	*otr1R(Sph1)::ura4⁺*	*dhp1-1*
SPJT42	*Mat1 Msmt0*	*leu1-32*	*ade6-216*	*his2*	*ura4 DS/E*	*otr1R(Sph1)::ura4⁺*	*dhp1-2*
SPKR1	*Mat1 Msmt0*	*leu1-32*	*ade6 DN/N*	*plus*	*ura4 DS/E*	*otr1R(Sph1)::ura4⁺, mat2::ade6⁺*	*dhp1-1*
SPJT191	*Mat1 Msmt0*	*leu1-32*	*ade6-210*	*plus*	-	*ura4::[CenH3.6KB+ade6⁺]*	*dhp1-1*
SPJT186a	*Mat1 Msmt0*	*leu1-32*	*ade6-210*	*his2*	-	*kΔ::ade6⁺ (off)*	*dhp1-1*
P1088	*Mat1 Msmt0*	*leu1-32*	*ade6 DN/N*	*plus*	*ura4 DS/E*	*otr1R(Sph1)::ura4⁺, mat2::ade6⁺*	*dhp1-1*
P1496	*h⁺*	*leu1-32*	*ade6⁺*	*plus*	*ura4-D18*	-	*dhp1-1, rpb2-flag-HTpA*
SPJT9	*h⁺*	*leu1-32*	*ade6-210*	*plus*	*ura4 DS/E*	*otr1R(Sph1)::ura4⁺*	*dhp1-FTP::NATN2*
SPWB6	*Mat1 Msmt0*	*leu1-32*	*ade6-216*	*his2*	*ura4 DS/E*	*otr1R(Sph1)::ura4⁺*	*din1Δ::kanMX*
SPWF39a	*Mat1 Msmt0*	*leu1⁺*	*ade6 DN/N*	*plus*	*ura4 DS/E*	*otr1R(Sph1)::ura4⁺, mat2::ade6⁺*	*din1Δ::kanMX*
SPJT240	*Mat1 Msmt0*	*leu1-32*	*ade6 DN/N*	*plus*	*ura4 DS/E*	*otr1R(Sph1)::ura4⁺, mat2::ade6⁺*	*din1Δ::kanMX*
p1040	*h⁺*	*leu1-32*	*ade6-210/216*	*plus*	*ura4-D18*	-	*din1Δ::kanMX, rpb2-flag-HTpA*
SPWB9	*Mat1 Msmt0*	*leu1-32*	*ade6-216*	*his2*	*ura4 DS/E*	*otr1R(Sph1)::ura4⁺*	*din1-FTP::NATN2*
SPJT23	*Mat1 Msmt0*	*leu1-32*	*ade6-216*	*his2*	*ura4 DS/E*	*otr1R(Sph1)::ura4⁺*	*ago1Δ::kanMX*
SPJT193a	*Mat1 Msmt0*	*leu1-32*	*ade6-216*	*his2*	-	*ura4::[CenH3.6KB+ade6⁺]*	*ago1Δ::kanMX*
SPJT249a	*Mat1 Msmt0*	*leu1-32*	*ade6 DN/N*	*plus*	*ura4 DS/E*	*otr1R(Sph1)::ura4⁺, mat2::ade6⁺*	*ago1Δ::kanMX*
SPJT132	*Mat1 Msmt0*	*leu1-32*	*ade6-210*	*his2*	*ura4 DS/E*	*otr1R(Sph1)::ura4⁺*	*ago1Δ::kanMX, dhp1-1*
SPJT242a	*Mat1 Msmt0*	*leu1-32*	*ade6 DN/N*	*plus*	*ura4 DS/E*	*otr1R(Sph1)::ura4⁺, mat2::ade6⁺*	*ago1Δ::kanMX, dhp1-1*
SPJT213a	*Mat1 Msmt0*	*leu1-32*	*ade6-216*	*his2*	*ura4 DS/E*	*otr1R(Sph1)::ura4⁺*	*ago1Δ::kanMX, rrp6Δ::kanMX*
SPKZ364	*Mat1 Msmt0*	*leu1-32*	*ade6-210*	*his2*	*ura4 DS/E*	*otr1R(Sph1)::ura4⁺*	*clr3Δ::kanMX*

S1 Table: List of strains used in this study (2 of 3)

Strains	*mat*	*leu1*	*ade6*	*his2*	*ura4*	Epigenetic Reporter	Mutation
SPJT122	*Mat1 Msmt0*	*leu1-32*	*ade6-210*	*his2*	*ura4 DS/E*	*otr1R(Sph1)::ura4*$^+$	*clr3Δ::kanMX, dhp1-1*
SPKZ569	*Mat1 Msmt0*	*leu1-32*	*ade6-210*	*plus*	*ura4 DS/E*	*otr1R(Sph1)::ura4*$^+$	*clr4Δ::kanMX*
SPJT97	*Mat1 Msmt0*	*leu1-32*	*ade6 DN/N*	*plus*	*ura4 DS/E*	*otr1R(Sph1)::ura4*$^+$, *mat2::ade6*$^+$	*clr4Δ::kanMX*
SPJT123a	*Mat1 Msmt0*	*leu1-32*	*ade6-210*	*his2*	*ura4 DS/E*	*otr1R(Sph1)::ura4*$^+$	*clr4Δ::kanMX, dhp1-1*
SPJT239a	*Mat1 Msmt0*	*leu1-32*	*ade6-216*	*his2*	*ura4 DS/E*	*otr1R(Sph1)::ura4*$^+$	*clr4Δ::kanMX, dcr1Δ::kanMX*
SPJT225	*Mat1 Msmt0*	*leu1-32*	*ade6-210*	*his2*	*ura4 DS/E*	*otr1R(Sph1)::ura4*$^+$	*clr4Δ::kanMX, dcr1Δ::kanMX, dhp1-1*
SPJT119a	*Mat1 Msmt0*	*leu1*$^+$	*ade6-210*	*his2*	*ura4 DS/E*	*otr1R(Sph1)::ura4*$^+$	*clr4Δ::kanMX, clr4*$^+$
SPJT133a	*Mat1 Msmt0*	*leu1*$^+$	*ade6-210*	*his2*	*ura4 DS/E*	*otr1R(Sph1)::ura4*$^+$	*clr4Δ::kanMX, clr4*$^+$*, dhp1-1*
SPJT251a	*Mat1 Msmt0*	*leu1*$^+$	*ade6-216*	*his2*	*ura4 DS/E*	*otr1R(Sph1)::ura4*$^+$	*clr4Δ::kanMX, clr4*$^+$*, dcr1Δ::kanMX*
p1495	*h*$^+$	*leu1-32*	*ade6*$^+$	*plus*	*ura4 DS/E*	*otr1R(Sph1)::ura4*$^+$	*clr4Δ::kanMX, rpb2-flag*-HTpA
SPJT231a	*Mat1 Msmt0*	*leu1*$^+$	*ade6-210*	*his2*	*ura4 DS/E*	*otr1R(Sph1)::ura4*$^+$	*clr4Δ::kanMX, clr4*$^+$*, dcr1Δ::kanMX, dhp1-1*
SPHC272	*Mat1 Msmt0*	*leu1-32*	*ade6-216*	*his2*	*ura4 DS/E*	*otr1R(Sph1)::ura4*$^+$	*dcr1Δ::kanMX*
SPWF1	*Mat1 Msmt0*	*leu1-32*	*ade6-210*	*his2*	*ura4 DS/E*	*otr1R(Sph1)::ura4*$^+$	*dcr1Δ::kanMX, dhp1-1*
SPWF33	*Mat1 Msmt0*	*leu1-32*	*ade6-210*	*his2*	*ura4 DS/E*	*otr1R(Sph1)::ura4*$^+$	*epe1Δ::kanMX*
SPJT44	*Mat1 Msmt0*	*leu1-32*	*ade6-216*	*his2*	*ura4 DS/E*	*otr1R(Sph1)::ura4*$^+$	*dhp1-1, epe1Δ::kanMX*
SPJT166a	*Mat1 Msmt0*	*leu1-32*	*ade6-210*	*his2*	*ura4-D18*	*otr1R(Sph1)::ade6*$^+$	*rhn1Δ::kanMX*
SPJT167a	*Mat1 Msmt0*	*leu1-32*	*ade6-210*	*his2*	*ura4 DS/E*	*otr1R(Sph1)::ura4*$^+$	*dhp1-1, rhn1Δ::kanMX*
SPKZ1153	*Mat1 Msmt0*	*leu1-32*	*ade6-216*	*his2*	*ura4 DS/E*	*otr1R(Sph1)::ura4*$^+$	*rrp6Δ::kanMX*
SPJT169b	*Mat1 Msmt0*	*leu1-32*	*ade6-210*	*his2*	*ura4 DS/E*	*otr1R(Sph1)::ura4*$^+$	*dhp1-1, rrp6Δ::kanMX*
SPKZ841	*Mat1 Msmt0*	*leu1-32*	*ade6-210*	*his2*	*ura4 DS/E*	*otr1R(Sph1)::ura4*$^+$	*rpb2-m203*
SPCY6a	*Mat1 Msmt0*	*leu1-32*	*ade6-210*	*his2*	*ura4 DS/E*	*otr1R(Sph1)::ura4*$^+$	*dhp1-1, rpb2-m203*
SPJT72	*Mat1 Msmt0*	*leu1-32*	*ade6-216*	*his2*	*ura4-D18*	*otr1R(Sph1)::ura4*$^+$	*sir2Δ::kanMX*
SPJT138	*Mat1 Msmt0*	*leu1-32*	*ade6 DN/N*	*his2*	*ura4 DS/E*	*otr1R(Sph1)::ura4*$^+$	*dhp1-1, sir2Δ::kanMX*
SPKZ392	*Mat1 Msmt0*	*leu1-32*	*ade6-210*	*his2*	*ura4 DS/E*	*otr1R(Sph1)::ura4*$^+$	*swi6Δ::NatN2*
SPJT164b	*Mat1 Msmt0*	*leu1-32*	*ade6-210*	*his2*	*ura4 DS/E*	*kΔ::ade6*$^+$ (off)	*swi6Δ::NatN2*

S1 Table: List of strains used in this study (3 of 3)

Strains	*mat*	*leu1*	*ade6*	*his2*	*ura4*	Epigenetic Reporter	Mutation
SPT783	*Mat1 Msmt0*	*leu1-32*	*ade6-216*	*his2*	*ura4 DS/E*	*otr1R(Sph1)::ura4*$^{+}$	*mit1-myc::kanMX*
SPJT274a	*Mat1 Msmt0*	*leu1-32*	*ade6-210*	*his2*	*ura4 DS/E*	*otr1R(Sph1)::ura4*$^{+}$	*mit1-myc::kanMX, dhp1-1*
SPJT276	*Mat1 Msmt0*	*leu1-32*	*ade6-216*	*his2*	*ura4 DS/E*	*otr1R(Sph1)::ura4*$^{+}$	*mit1-myc::kanMX, din1Δ::kanMX*
SPJT278a	*Mat1 Msmt0*	*leu1-32*	*ade6-216*	*his2*	*ura4 DS/E*	*otr1R(Sph1)::ura4*$^{+}$	*mit1-myc::kanMX, clr4Δ::kanMX*
SPJT292a	*Mat1Msmt0*	*leu1-32*	*ade6-DN/N*	*plus*	*ura4 DS/E*	*otr1R(Sph1)::ura4*$^{+}$*, mat2::ade6*$^{+}$	*+ pREP41 (vector)*
SPJT288a	*Mat1Msmt0*	*leu1-32*	*ade6-DN/N*	*plus*	*ura4 DS/E*	*otr1R(Sph1)::ura4*$^{+}$*, mat2::ade6*$^{+}$	*dhp1-1 + pREP41 (vector)*
SPJT293	*Mat1Msmt0*	*leu1-32*	*ade6-DN/N*	*plus*	*ura4 DS/E*	*otr1R(Sph1)::ura4*$^{+}$*, mat2::ade6*$^{+}$	*+ pdhp1*$^{+}$
SPWF76a	*Mat1 Msmt0*	*leu1-32*	*ade6-210*	*his2*	*ura4 DS/E*	*otr1R(Sph1)::ura4*$^{+}$	*dhp1-1 + pdhp1*$^{+}$
SPJT301a	*Mat1Msmt0*	*leu1-32*	*ade6-DN/N*	*plus*	*ura4 DS/E*	*otr1R(Sph1)::ura4*$^{+}$*, mat2::ade6*$^{+}$	*+ pdhp1-D55A E207Q*
SPJT303a	*Mat1Msmt0*	*leu1-32*	*ade6-DN/N*	*plus*	*ura4 DS/E*	*otr1R(Sph1)::ura4*$^{+}$*, mat2::ade6*$^{+}$	*dhp1-1 + pdhp1-D55A E207Q*
