# Supplementary material for: A Novel Epigenetic Silencing Pathway Involving the Highly Conserved 5’-3’ Exoribonuclease Dhp1/Rat1/Xrn2 in Schizosaccharomyces pombe
Source: PLoS Genet. 2016 Feb 18;12(2):e1005873. doi: 10.1371/journal.pgen.1005873 (PMC4758730; doi:10.1371/journal.pgen.1005873)
Supplement: S2 Table — (PDF) [file pgen.1005873.s002.pdf]

S2 Table: Relative Expression of Silencing Factors*

	dhp1-1/WT	din1Δ/WT
swi6	0.854685647	1.136978862
chp2	0.98005497	1.321010606
clr4	0.968112804	0.966577954
rik1	0.947395296	1.155050305
stc1	1	1.183624876
pcu4 (cul4)	1	1.122932913
raf1 (dos1)	0.957442401	1.404922713
raf2 (dos2)	0.99886828	1.121689036
clr1	0.970989556	1.044235404
clr2	0.564879803	0.907492358
clr3	0.758853321	0.938691001
mit1	0.96593702	1.077740887
ccq1	0.952139665	1.029906927
taz1	1.085419344	1.128192968
epe1	1	0.864043559
sir2	1.051220944	1.146409381
cdc20	0.987164085	1.157919748
atf1	0.76339746	0.850402544
seb1	1.312382603	0.722538992
red1	1.135230283	1
mmi1	0.924895086	0.844050436
air1	1.047961649	1.027582095
pcr1	1	0.508359146
rrp6	1.142199137	1
cid14	1	1.479324985
ago1	1.041339871	1.155622389
chp1	0.90897002	0.772592003
tas3	0.771002973	1.123871657
dcr1	0.758263135	1.00413756
rdp1	1	1.205329488
grc3	1	0.962710159
ipi1	1.082023239	1.48851199
crb3	0.782176827	1.173931179
rix1	1	1.379565988
las1	1	1.128689858
act1	0.892686966	0.804520339

* Reported values are average of all probes covering the coding region of the specified gene normalized to wildtype.
