# Supplementary material for: A Novel Epigenetic Silencing Pathway Involving the Highly Conserved 5’-3’ Exoribonuclease Dhp1/Rat1/Xrn2 in Schizosaccharomyces pombe
Source: PLoS Genet. 2016 Feb 18;12(2):e1005873. doi: 10.1371/journal.pgen.1005873 (PMC4758730; doi:10.1371/journal.pgen.1005873)
Supplement: S3 Table — (PDF) [file pgen.1005873.s003.pdf]

S3 Table: Dhp1-FTP- and Din1-FTP-interacting proteins identified by LC-MS/MS*

Name	Description	Dhp1-FTP Purification Abundance** (% of bait)	Din1-FTP Purification Abundance** (% of bait)
Dhp1	5'-3' exoribonuclease	100	34.75
Din1	Dhp1p-interacting protein	50.47	100
Mug161	CwfJ family protein, splicing factor (predicted)	36.55	6.25
Sum3	Translation initiation RNA helicase (Moc2)	12.62	4.1
Spt5	Transcription elongation factor	8.64	5.63
Htb1	Histone H2B	5.77	0.70
Rpb2	RNA polymerase II complex subunit	5.65	6.95
Rpb1	RNA polymerase II large subunit	5.43	11.17
Hta1	histone H2A alpha	4.57	0.062
Rpb7	DNA-directed RNA polymerase complex II subunit	4.51	9.76
Rpb5	DNA-directed RNA polymerase I, II and III subunit	4.14	3.95
Srp2	mRNA export factor	3.78	0.55
Rpb9	DNA-directed RNA polymerase II complex subunit	2.46	2.11
Mlo3	RNA binding protein Mlo3	1.23	0.05
Mmi1	YTH family RNA binding protein	0.50	0.014
→ Rrp6	Exosome 3'-5' exoribonuclease subunit	0.042	0
→ Rik1	Silencing Protein – ClrC subunit	0.017	0

*All proteins listed were not detected in the purification of the untagged control strain
**Abundance values for each identified protein are normalized to the bait values
