# Supplementary material for: A Novel Epigenetic Silencing Pathway Involving the Highly Conserved 5’-3’ Exoribonuclease Dhp1/Rat1/Xrn2 in Schizosaccharomyces pombe
Source: PLoS Genet. 2016 Feb 18;12(2):e1005873. doi: 10.1371/journal.pgen.1005873 (PMC4758730; doi:10.1371/journal.pgen.1005873)
Supplement: S4 Table — (PDF) [file pgen.1005873.s004.pdf]

S4 Table: List of oligonucleotides used in this study (1 of 3)

Primer	Sequence	Purpose
Rat1 FW 1	CGGAAAGATGGCACATGG	*dhp1* genotyping
Rat1 RV 3	ATGTTCGTTGCTTGCTGTGC	*dhp1* genotyping
Dhp1-1 FW 1	CAACCCTTCAAGCCTTACGA	*dhp1-1>>ura4* genotyping, tagging *dhp1/dhp1-1*
Ura 5' RV 1	TAGCCAGTGGGATTTGTAGC	*dhp1-1>>ura4* genotyping
Din1 UP F	TCCCAACCTGCATTACGTTG	*din1* genotyping, tagging din1
Din1 RV 1	CGACAGGATCAGCGTTCG	*din1* genotyping
Rrp6 3' FW	GACCTCTCTATAGTTGCCTG	*rrp6* genotyping
Rrp6 3' RV	TACTTAGCGTTTCCTCGCAG	*rrp6* genotyping
Ago1_frw_check	ATGCGGTAATTTAAGAAAAGC	*ago1Δ* genotyping
Ago1_rev_check	TCTGTATTTCTATATTGGCG	*ago1Δ* genotyping
Ago1 Inside F		*ago1* genotyping
Ago1 Inside R		*ago1* genotyping
Clr4 UP	TTGCTCTGAAATTGAACACATCGAC	*clr4* genotyping
Clr4 DOWN	CCGAGTCGGAGTTACTTCCCTTTAGTC	*clr4* genotyping
Clr4 RV 3	CTTCCTGATATAGCACTTCCC	*clr4Δ* genotyping
Dcr1 FW 1	CCTATACTGATGGACATGGG	*dcr1Δ* genotyping, amplify dcr1Δ::kanMX cassette
Dcr1 RV 2	TTGCTGGAATACGGTTGCAG	*dcr1Δ* genotyping
Swi6 UP	GTGATGCTAGCCATTCTGTAC	*swi6* genotyping
Swi6 DOWN	AGAGTCGCTTTATGAGAAGC	*swi6* genotyping
Swi6 RV 3	CTGATGTCTTCATGTGTTGC	*swi6Δ* genotyping
Clr3 RV 2	CACGTGCTAACCATTACACC	*clr3* genotyping
Epe1 RV 2	GTGTGTTGTAGTATCCGGCT	*epe1Δ* genotyping
Rhn1 RV 2	CTGCACAACACATGGATGCG	*rhn1Δ* genotyping
Sir2 RV 3	GGTTGTGGTTTAGGTTCTCG	*sir2* genotyping
Kan2	ATCATTGGCAACGCTACCTT	*KanMX6* genotyping (5' RV)
Kan3	GCGCAATCACGAATGAATAA	*KanMX6* genotyping (3' FW)
Nat2	GGTGTCGGTGGTGAAGGACC	*NatN2* genotyping (5' RV)
Nat3	TGCCCTGCCCCTAATCTCGA	*NatN2* genotyping (3' RV)
MT1	AGAAGAGAGAGTAGTTGAAG	Mating-type genotyping
MP	ACGGTAGTCATCGGTCTTCC	Mating-type genotyping
MM	TACGTTCAGTAGACGTAGTG	Mating-type genotyping
Ura4 DS/E #1	GAGGGGATGAAAAATCCCAT	*DS/E* genotyping, $ura4^+$ versus *DS/E* competitive PCR
Ura4 DS/E #2	TTCGACAACAGGATTACGACC	*DS/E* genotyping, $ura4^+$ versus *DS/E* competitive PCR
Ade6 #1	TGCGATGCACCTGACCAGGAAAGT	*DN/N* genotyping, $ade6^+$ versus *DN/N* competitive PCR
Ade6 #2	AGAGTTGGGTGTTGATTTCGCTGA	*DN/N* genotyping, $ade6^+$ versus *DN/N* competitive PCR
JPO4	CGTGAGTATACAAACAAATACACTAGG	*otr::ura4* genotyping
JPO17	CTACTCTTCTCGATGATCCTGTAA	*otr::ura4* genotyping
Ade6 3' FW 1	GGCGACCATAGACATAACTG	$K\Delta::ade6^+$ genotyping
K RV 2	GGTTTGTTTAGCGCACTTTG	$K\Delta::ade6^+$ genotyping

S4 Table: List of oligonucleotides used in this study (2 of 3)

Primer	Sequence	Purpose
Kan_Fwd	CGGATCCCCGGGTTAATTAA	Amplify *KanMX6* Cassette
Kan_Rev	GAATTCGAGCTCGTTTAAAC	Amplify *KanMX6* Cassette
Nat-F2	CGGATCCCCGGGTTAATTAA	Amplify *NatN2* Cassette
Nat2-S2	ATCGATGAATTCGAGCTCG	Amplify *NatN2* Cassette
Ade6 Full FW	TATGCCTTTCGATGCCAATTG	Amplify *ade6*$^{+}$ Cassette
Ade6 Full RV	GAACATGGTCAACGGGG	Amplify *ade6*$^{+}$ Cassette
Clr3 FW 1	CGTCATTCACTCGTGTTTGT	Amplify *clr3Δ::kanMX* Cassette
Clr3 RV 1	TGGATGAGGGATTTCCCTCA	Amplify *clr3Δ::kanMX* Cassette
Clr4 F1	ACACAGATGGGTTAGACTCC	Amplify *clr4Δ::kanMX* Cassette
Clr4 R1	CAGGCAGATCCTTCCCTTGA	Amplify *clr4Δ::kanMX* Cassette
Dcr1 RV 1	GGAAGCTCAATCAATCGACG	Amplify *dcr1Δ::kanMX* Cassette
Epe1 FW 1	GCCGAATATAGCCTGCTTCA	Amplify *epe1Δ::kanMX* Cassette
Epe1 RV 1	CGAGTCAAAGTGGATTGATGC	Amplify *epe1Δ::kanMX* Cassette
K FW	CCGAATACCACGAAGTTATTG	Amplify K region fragment 1 (*KΔ::ade6*$^{+}$)
Ade6-KΔ R	CAATTGGCATCGAAAGGCATAGTTCAG ACTGATCTCGCAATT	Amplify K region fragment 1 (*KΔ::ade6*$^{+}$)
Ade6-KΔ F	AAACCCCGTTGACCATGTTCGCTTTCC TCTCAGCATTCAG	Amplify K region fragment 3 (*KΔ::ade6*$^{+}$)
K RV	GATCGCCAAAGTTGTATTGC	Amplify K region fragment 3 (*KΔ::ade6*$^{+}$)
Rhn1 FW 1	TCGTTTGCGATGCTAACAGC	Amplify *rhn1Δ::kanMX* cassette
Rhn1 RV 1	GCATATCCTCTACTTCTGCC	Amplify *rhn1Δ::kanMX* cassette
Swi6 5' FW	CTTGAGAGACTCAGCATTGC	Amplify *swi6Δ::NATN2* cassette
Swi6 RV 1	CATGAAGAGCAGTGCATGC	Amplify *swi6Δ::NATN2* cassette
Nat_Rat1_R	TTAATTAACCCGGGGATCCGATAGTAGC CATTCCTGTTATTAT	*dhp1* tagging
Nat_Rat1_F	CGAGTCGAATTCATCGATGCGGGTTTG GGATATTGGAG	*dhp1* tagging
Rat1_dw_R	GGGGACATTCTACTGTTGTAG	*dhp1* tagging
Kan_Din_R	TTAATTAACCCGGGGATCCGAATTATTT GTCGTAAAACAAG	*din1* tagging
Kan_Din_F	CGAGCTCGAATTCATCGATAGTAGGGT TATACACTAGATGT	*din1* tagging
Din DOWN R	CAACCACGCCCTCCATATAC	*din1* tagging
Sir2 FW 1	ACGCATTCCAGTTCTCATGG	*sir2* deletion
Sir2 FW 2	CCTCCTACTACTCCTCAGAT	*sir2* tagging
NAT Sir2 FW	CGAGCTCGAATTCATCGATGGACTGAT CGATCTATCGAA	*sir2* deletion/tagging
NAT Sir2 RV 1	TTAATTAACCCGGGGATCCGATAGACTT GCGCGTACCCTT	*sir2* deletion
NAT Sir2 RV 2	TTAATTAACCCGGGGATCCGACAACTA CCGTTTGTTGTGC	*sir2* tagging
Sir2 RV 1	ACCTTTTGGCATGCTCGATG	*sir2* deletion/tagging

S4 Table: List of oligonucleotides used in this study (3 of 3)

Primer	Sequence	Purpose
q-Actin F	GGTTTCGCTGGAGATGATG	qRT-PCR: *act1*$^{+}$ (Endogenous Control)
q-Actin R	ATACCACGCTTGCTTTGAG	qRT-PCR: *act1*$^{+}$ (Endogenous Control)
q-Leu1 F1	ATTGCTCGTTTGGCTGCTTG	qChIP: *leu1*$^{+}$ (Endogenous Control)
q-Leu1 R1	TACAGTCTTGCGCCAAAGAC	qChIP: *leu1*$^{+}$ (Endogenous Control)
q-Ade6 F	ATGCTTATCCTACAACTGAGACC	qPCR: a*de6*$^{+}$
q-Ade6 R	TGAATTGAGAAGGGAAGACGAG	qPCR: a*de6*$^{+}$
q-Cen(dg) F	AATTGTGGTGGTGTGGTAATAC	qPCR: Centromere (*dg*)
q-Cen(dg) R	GGGTTCATCGTTTCCATTCAG	qPCR: Centromere (*dg*)
q-Mat F3	AGTAATTCTATGTACCCGTGC	qPCR: Mating-type locus (*cenH*)
q-Mat R3	CGTCGTCACCAATCGATCTT	qPCR: Mating-type locus (*cenH*)
qUra4 5’ F1	CTAGATTCTAGGATGCGTATTGG	qPCR: Spreading Assay "A"
qUra4 5’ R1	CAGTTTGCTCATGGGTACAAC	qPCR: Spreading Assay "A"
Ura4 StuI F1	AATTGCCATACAGTGCCAGG	qPCR: Spreading Assay "B"
q-CenH ade6 FW 2	GGTTAAATTGTGGTGGTGTGG	qPCR: Spreading Assay "B"
q-CenH ade6 FW 1	TGACAAAGGTGCCGAATCAAC	qPCR: Spreading Assay "C"
q-CenH ade6 RV 1	TTGCAACAGGCGGCCAAGC	qPCR: Spreading Assay "C"
qUra4 3’ F1	CTGGCAATGTTACGAGGAATC	qPCR: Spreading Assay "D"
qUra4 3’ R1	TTCAGAAGAACATTACTCGTCG	qPCR: Spreading Assay "D"
Dhp1 D55A FW	GACAACTTGTATTTGGCCATGAATGG TATTGTTCATC	*dhp1* mutagenesis
Dhp1 D55A RV	GATGAACAATACCATTCATGGCCAAA TACAAGTTGTC	*dhp1* mutagenesis
Dhp1 E207Q FW	GTTCCTGGTGAAGGTCAACATAAGAT CATGGAATT	*dhp1* mutagenesis
Dhp1 E207Q RV	AATTCCATGATCTTATGTTGACCTTCA CCAGGAAC	*dhp1* mutagenesis
Pst1- Dhp1 FW	ATCGCTGCAGCGCTACCATTGATGA GTCTG	Clone fragment containing *dhp1*$^{+}$ gene into pREP41
BamHI-Dhp1 RV	TCTAGGATCCCTCGATTGATGGATCT TATGCATG	Clone fragment containing *dhp1*$^{+}$ gene into pREP41
