# Supplementary figures and images for: A Novel Epigenetic Silencing Pathway Involving the Highly Conserved 5’-3’ Exoribonuclease Dhp1/Rat1/Xrn2 in Schizosaccharomyces pombe
Source: PLoS Genet. 2016 Feb 18;12(2):e1005873. doi: 10.1371/journal.pgen.1005873 (PMC4758730; doi:10.1371/journal.pgen.1005873)

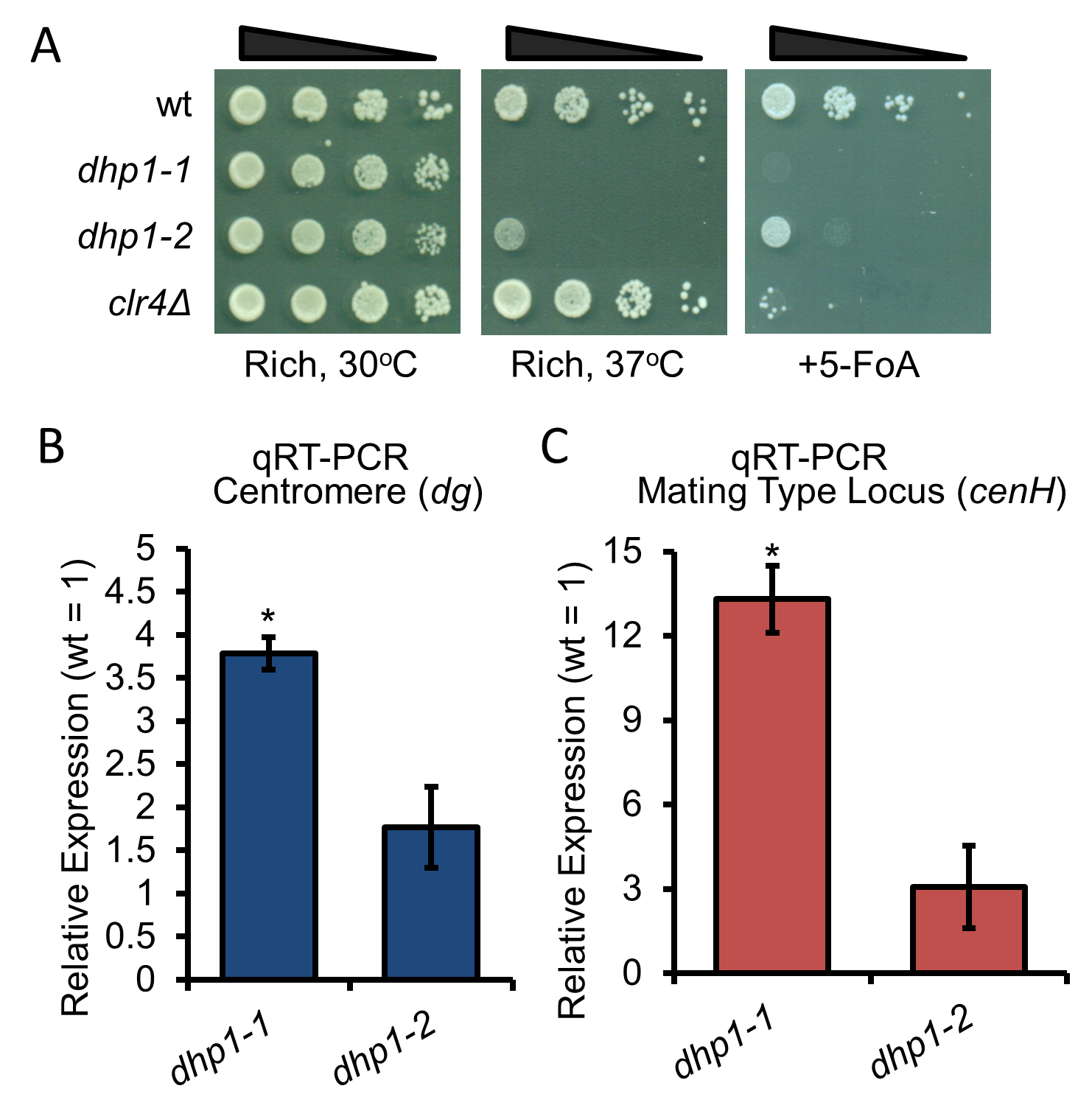

Supplement: S1 Fig — (A) Ten-fold serial dilutions demonstrate the expression of otr∷ura4+ (on 5-FoA-containing media) and the degree of centromeric heterochromatin functionality (on TBZ-containing media). (B-C) dhp1-1 has stronger silencing defect than dhp1-2 as evaluated by qRT-PCR at the centromeric region (B) and the mating type locus (C). * P ≤ 0.05 as determined by student’s t test comparing the indicated sample values with wt values. Error bar represent s.e.m. (TIF) [file pgen.1005873.s005.tif]

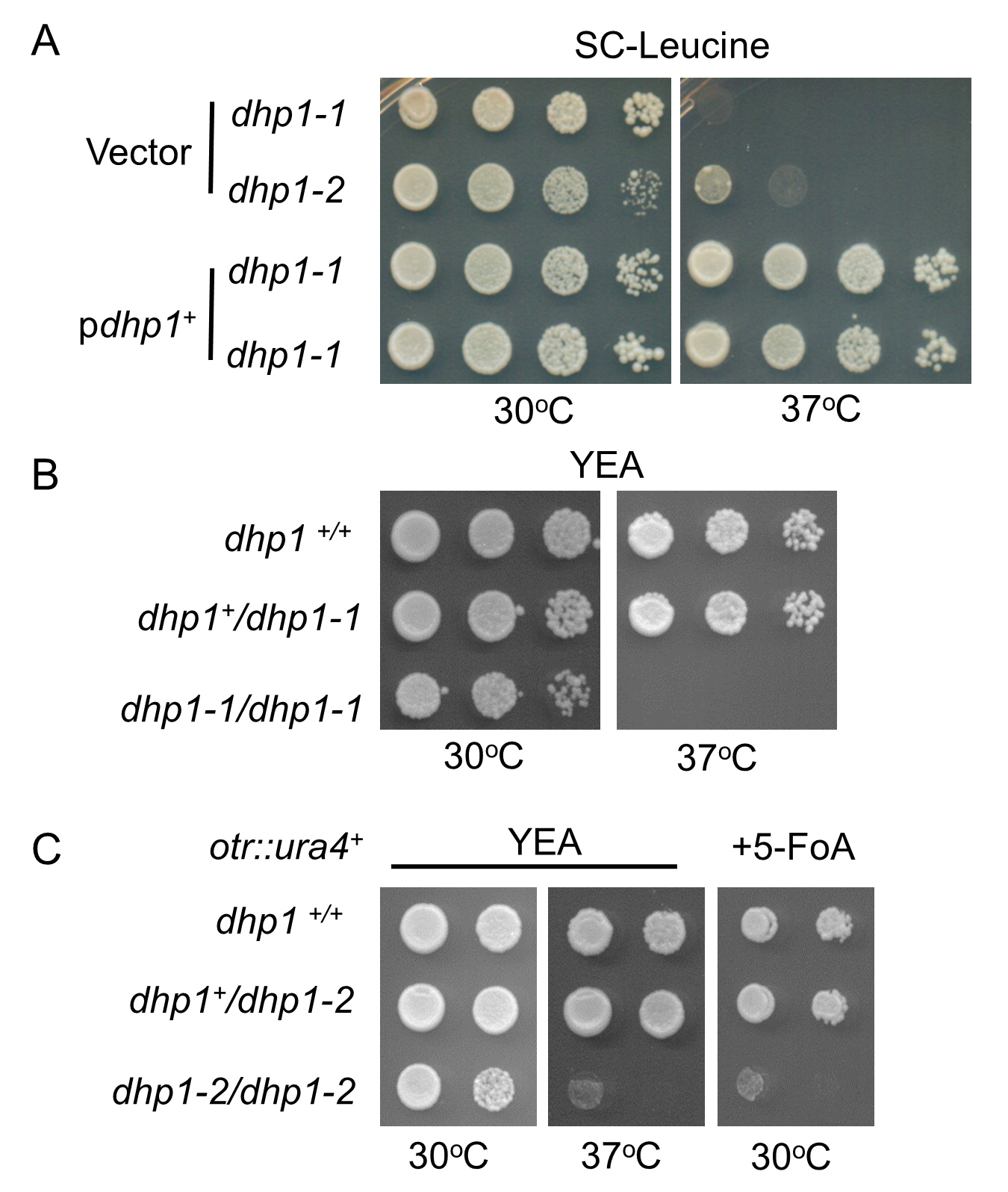

Supplement: S2 Fig — (A) dhp1-1 ts phenotype is rescued by a wildtype dhp1+ allele borne from a plasmid, pdhp1+. Two independent colonies for dhp1-1 or dhp1-2 carrying pdhp1+ are shown. All cells were cultured on SC-leucine medium. (B-C) Ten-fold serial dilutions show the growth of diploid wildtype (wt), dhp1-1 (B) or dhp1-2 (C) heterozygotes and homozygotes on rich medium at indicated temperatures. The expression of otr∷ura4+ in dhp1-2 is assayed on 5-FoA-containing counter-selective media at 30°C (C). (TIF) [file pgen.1005873.s006.tif]

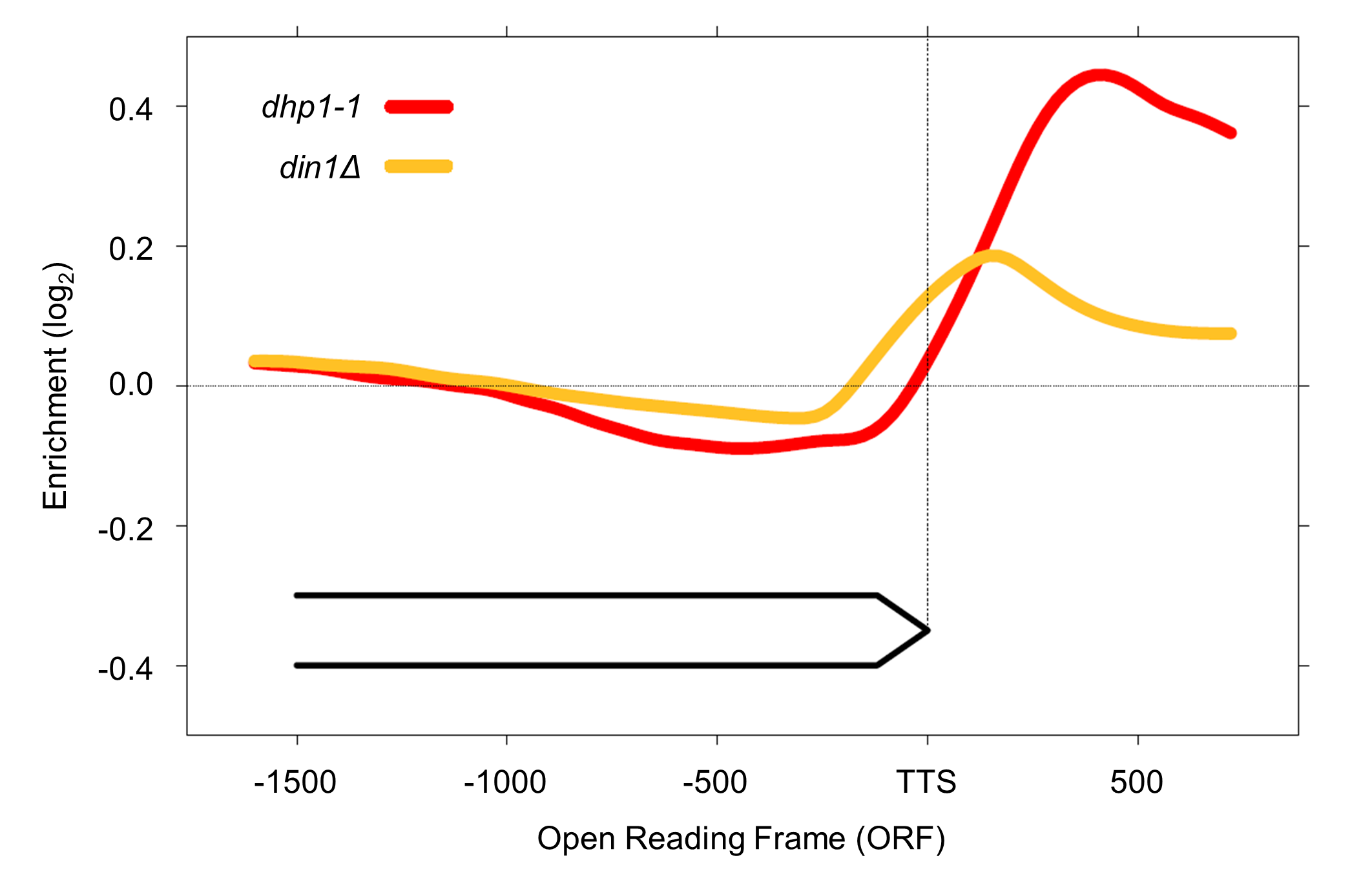

Supplement: S3 Fig — Expression profiling shows average RNA expression around transcriptional termination site (TTS), normalized to wildtype (Enrichment (log2)). dhp1-1 and din1Δ show increased RNA levels after the normal termination site, indicating a transcription termination defect in both although the defect is greater in dhp1-1. (TIF) [file pgen.1005873.s007.tif]

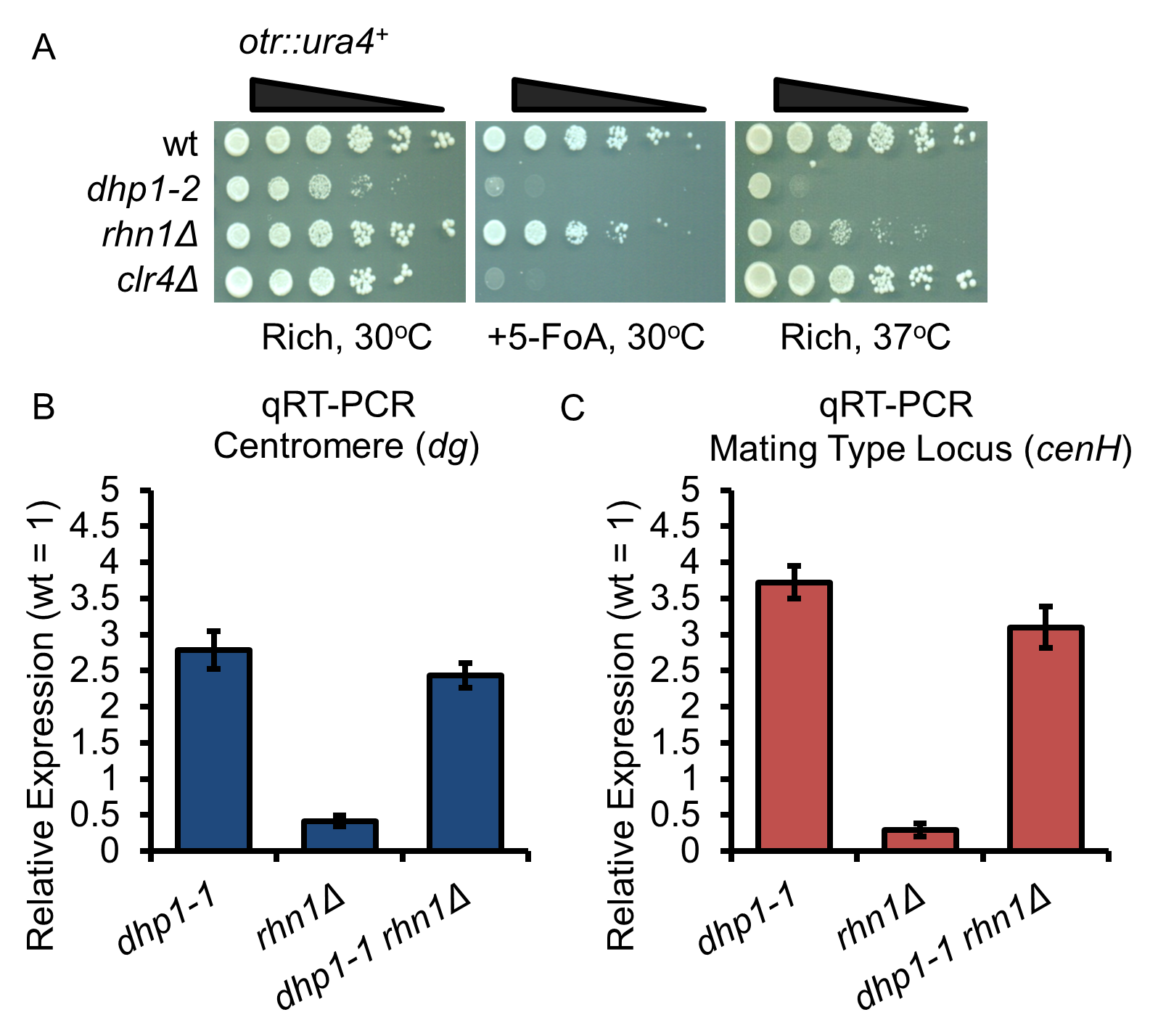

Supplement: S4 Fig — (A) Ten-fold serial dilution on plates containing indicated media and cultured at indicated temperature. (B-C) qRT-PCR demonstrates the silencing defect observed in dhp1-1. Only minor silencing defects can be detected in rhn1Δ by dilution assay, but no silencing defect is observed by qRT-PCR at the (B) centromere or (C) mating type locus. Error bar represent s.e.m. (TIF) [file pgen.1005873.s008.tif]

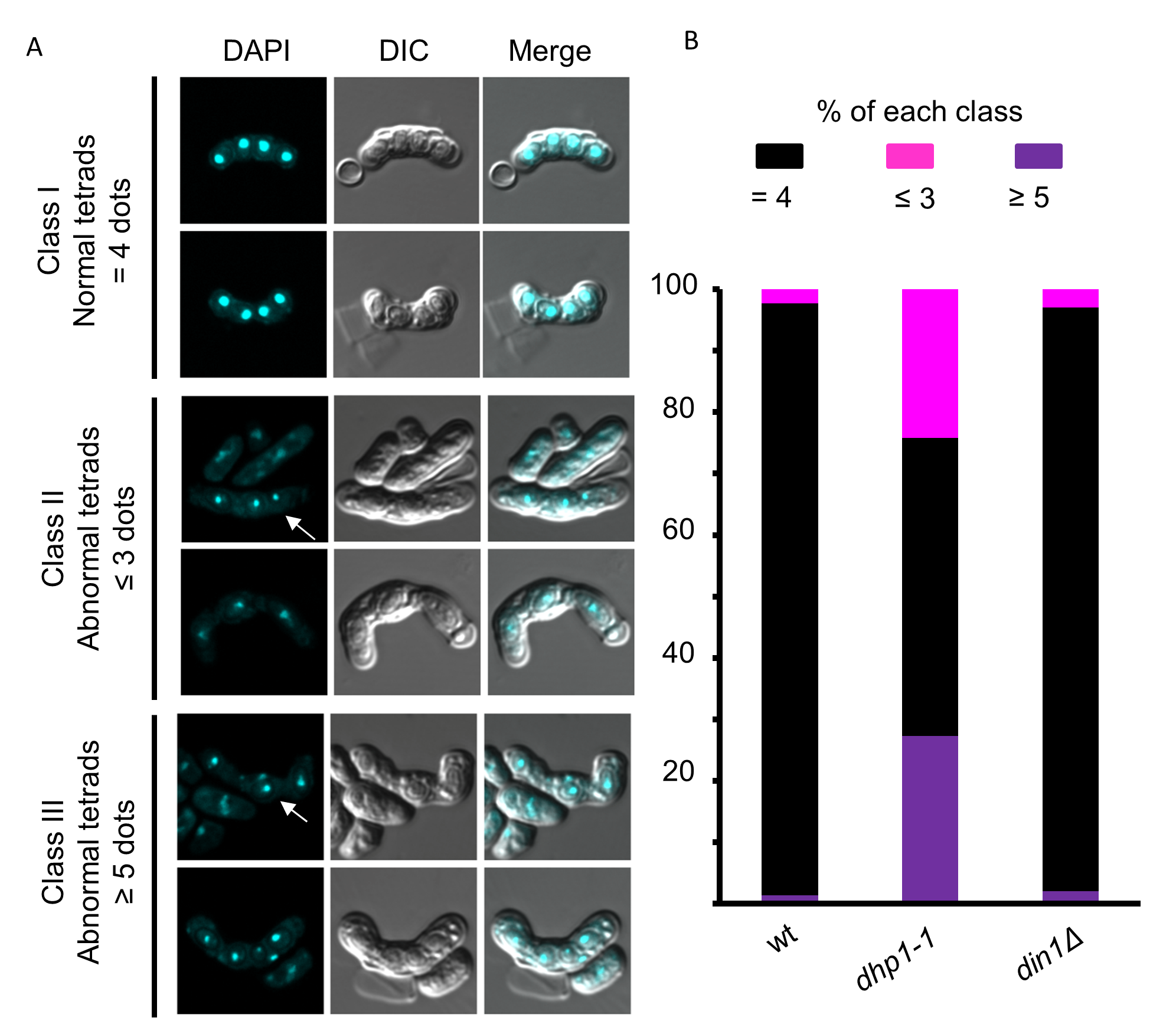

Supplement: S5 Fig — (A) Aberrant meiotic segregation as visualized by DAPI staining. Class I represents normal meiotic segregation, in which each spore from a tetrad receives a similar amount of DNA (blue dots). Both class II and class III represent mis-segregation events during meiotic division resulting in either ≤ 3 dots (class II) or ≥ 5 dots (class III). (B) The percentage of frequency with which the respective phenotypic classes were calculated. More than 150 randomly selected tetrads of each indicated strain were scored. (TIF) [file pgen.1005873.s009.tif]

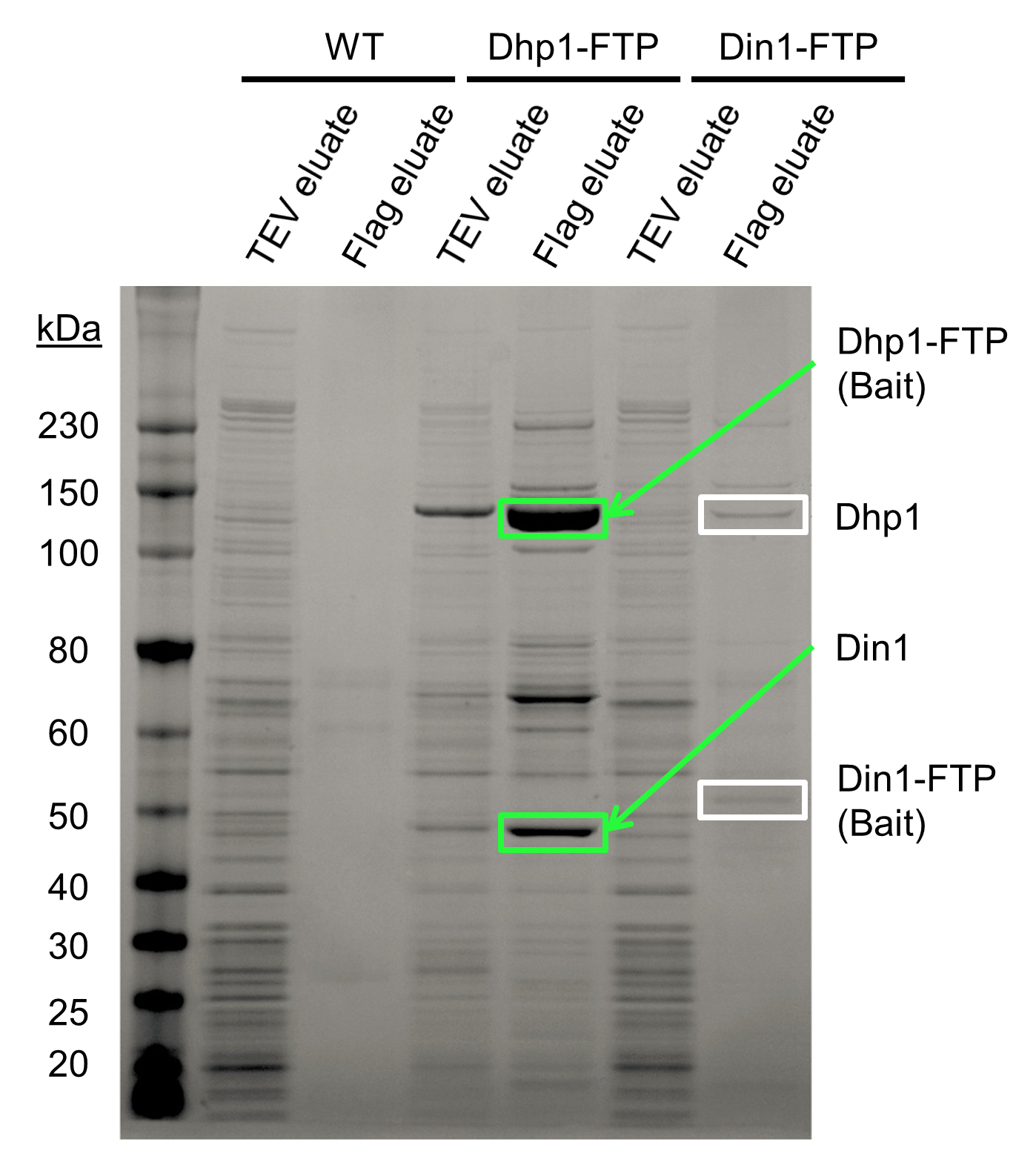

Supplement: S6 Fig — Brilliant blue stained 4–12% NuPAGE gel showing two-step affinity purification results for Dhp1-FTP and Din1-FTP. The bands shown here represent proteins which have direct physical interaction with the bait proteins, Dhp1-FTP or Din1-FTP, as purifications were carried out in the presence of Benzonase. (WT, untagged control strain; FTP, a Flag-Protein A tandem epitope tag). A list of peptides identified in the purified fractions by LC-MS/MS can be found in S3 Table. (TIF) [file pgen.1005873.s010.tif]

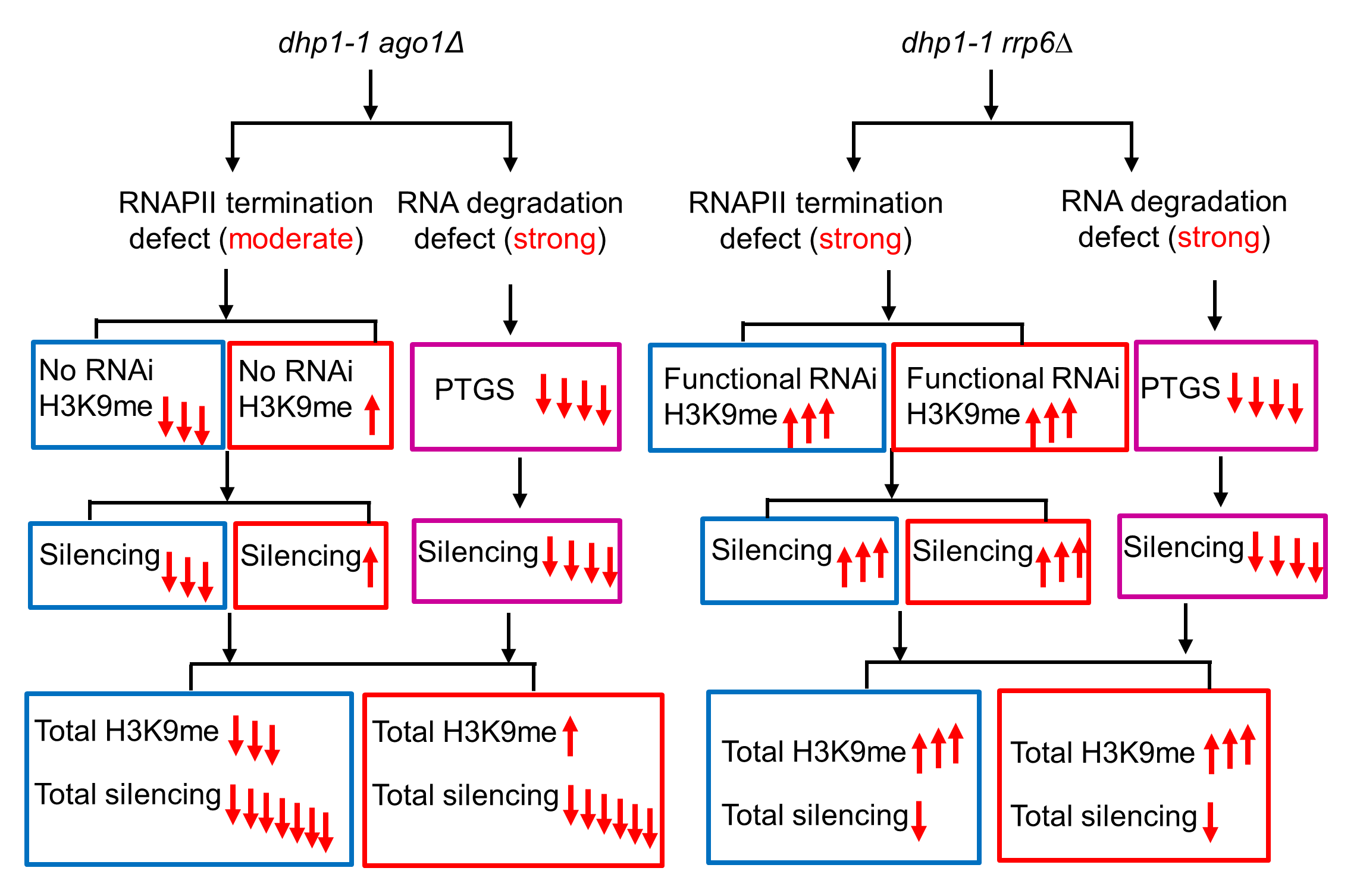

Supplement: S7 Fig — Blue boxes represent the effects of combined mutations at the centromeric region; red boxes represent these effects at the mating type locus; and purple boxes represent the overall effects at both loci. Red arrows depict the relative changes in H3K9me and silencing levels compared to wildtype. We propose that the expression of repeat transcripts and the level of H3K9me observed in dhp1-1 together with either ago1Δ or rrp6Δ are additive effects of impaired transcription termination and defective RNA degradation (PTGS). (TIF) [file pgen.1005873.s011.tif]

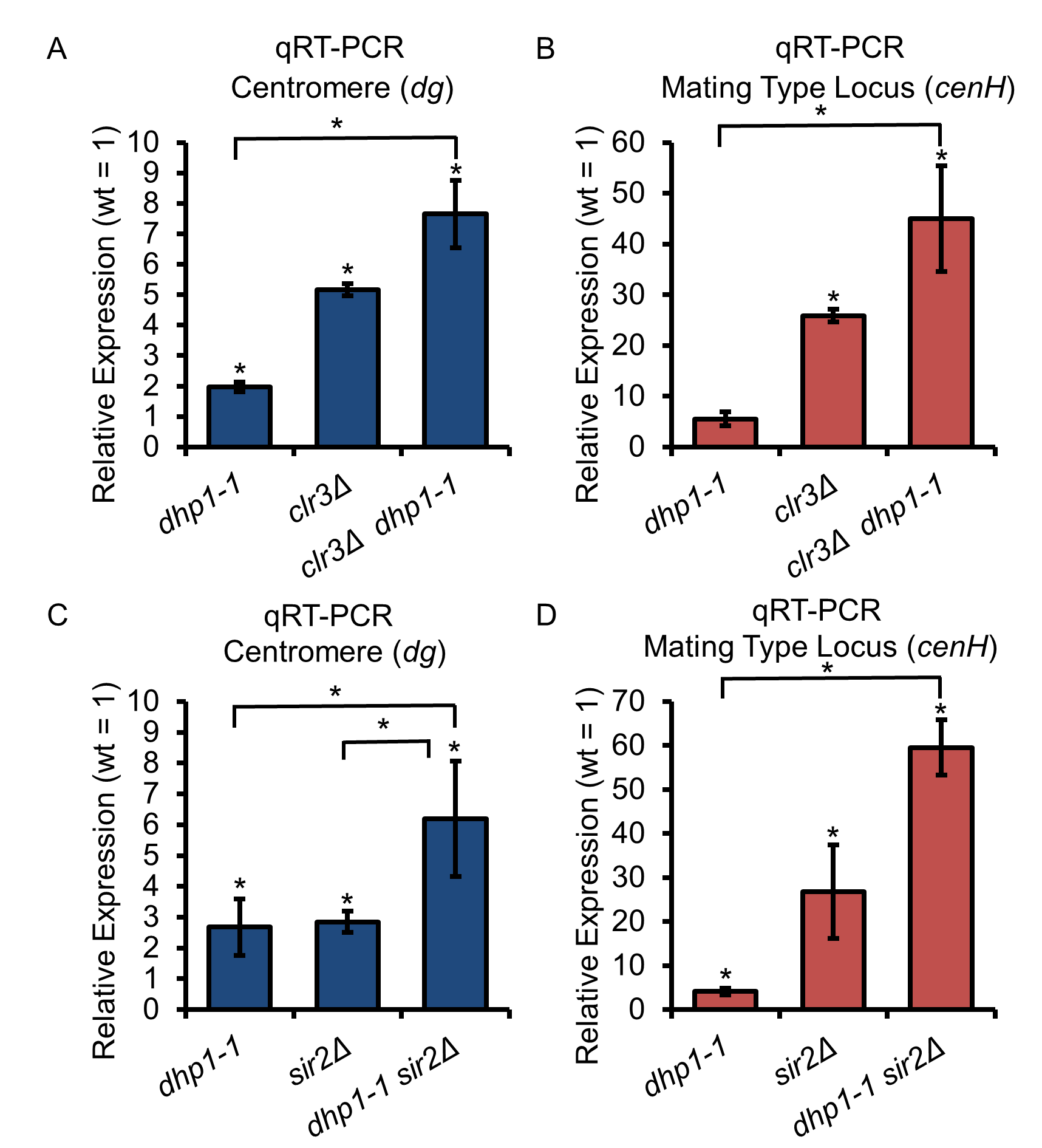

Supplement: S8 Fig — qRT-PCR analysis shows negative genetic interactions between dhp1-1 and clr3Δ (A-B) and sir2Δ (C-D) for the silencing of centromeric (A and C) and mating type locus repeats (B and D). *P ≤ 0.05 as determined by student’s t test comparing the indicated sample values with the wt. Significance between single mutants and double mutants are indicated by horizontal lines linking the compared samples together. (TIF) [file pgen.1005873.s012.tif]

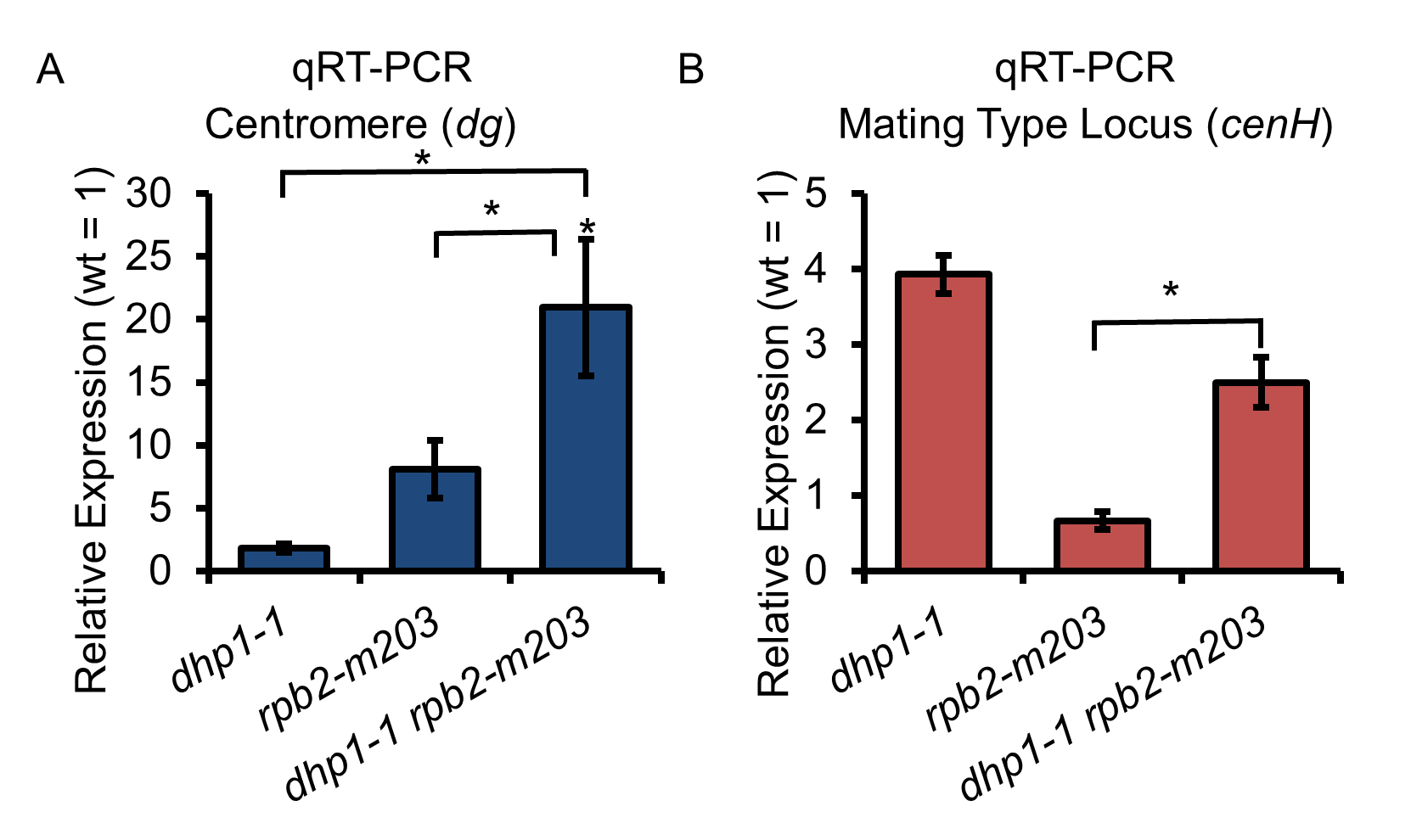

Supplement: S9 Fig — qRT-PCR analysis of silenced repeat regions in the (A) centromere and (B) mating type locus shows a negative genetic interaction between dhp1-1 and rpb2-m203. *P ≤ 0.05 as determined by student’s t test comparing the indicated sample values with the wt. Significance between single mutants and double mutants are indicated by horizontal lines linking the compared samples together. (TIF) [file pgen.1005873.s013.tif]
